# Detecting distant-homology protein structures by aligning deep neural-network based contact maps

**DOI:** 10.1371/journal.pcbi.1007411

**Published:** 2019-10-17

**Authors:** Wei Zheng, Qiqige Wuyun, Yang Li, S. M. Mortuza, Chengxin Zhang, Robin Pearce, Jishou Ruan, Yang Zhang

**Affiliations:** 1 Department of Computational Medicine and Bioinformatics, University of Michigan, Ann Arbor, MI, United States of America; 2 College of Mathematical Sciences and LPMC, Nankai University, Tianjin, PR China; 3 Computer Science and Engineering Department, Michigan State University, East Lansing, MI, United States of America; 4 State Key Laboratory of Medicinal Chemical Biology, Nankai University, Tianjin, PR China; 5 Department of Biological Chemistry, University of Michigan, Ann Arbor, MI, United States of America; University of Oxford, UNITED KINGDOM

## Abstract

Accurate prediction of atomic-level protein structure is important for annotating the biological functions of protein molecules and for designing new compounds to regulate the functions. Template-based modeling (TBM), which aims to construct structural models by copying and refining the structural frameworks of other known proteins, remains the most accurate method for protein structure prediction. Due to the difficulty in recognizing distant-homology templates, however, the accuracy of TBM decreases rapidly when the evolutionary relationship between the query and template vanishes. In this study, we propose a new method, CEthreader, which first predicts residue-residue contacts by coupling evolutionary precision matrices with deep residual convolutional neural-networks. The predicted contact maps are then integrated with sequence profile alignments to recognize structural templates from the PDB. The method was tested on two independent benchmark sets consisting collectively of 1,153 non-homologous protein targets, where CEthreader detected 176% or 36% more correct templates with a TM-score >0.5 than the best state-of-the-art profile- or contact-based threading methods, respectively, for the Hard targets that lacked homologous templates. Moreover, CEthreader was able to identify 114% or 20% more correct templates with the same Fold as the query, after excluding structures from the same SCOPe Superfamily, than the best profile- or contact-based threading methods. Detailed analyses show that the major advantage of CEthreader lies in the efficient coupling of contact maps with profile alignments, which helps recognize global fold of protein structures when the homologous relationship between the query and template is weak. These results demonstrate an efficient new strategy to combine *ab initio* contact map prediction with profile alignments to significantly improve the accuracy of template-based structure prediction, especially for distant-homology proteins.

## Author summary

Despite decades of effort in computational method development, template-based modeling (TBM) still remains the most reliable approach to high-resolution protein structure prediction. Previous studies have shown that the PDB library is complete for single-domain proteins and TBM is in principle sufficient to solve the structure prediction problem if the most similar structure in the PDB could be reliably identified and used as template for model reconstruction. But in reality, the success of TBM depends on the availability of closely-homologous templates, where its accuracy and reliability decrease sharply when the evolutionary relationship between query and template becomes more distant. We developed a new threading approach, CEthreader, which allows for dynamic programing alignments of predicted contact-maps through eigen-decomposition. The large-scale benchmark tests show that the coupling of contact map with profile and secondary structure alignments through the proposed protocol can significantly improve the accuracy of template recognition for distantly-homologous protein targets.

This is a *PLoS Computational Biology* Methods paper.

## Introduction

Given the rapidly increasing gap between the number of known protein sequences and the number of known structures, the demand for high-resolution, computer-based protein structure prediction has risen dramatically [1,2]. While various advanced approaches have been proposed over the last few decades [3–8], template-based modeling (TBM), which is designed to construct structural models using templates of similar folds collected from the Protein Data Bank (PDB), remains still the most accurate and reliable protein structure prediction method [9,10]. The core procedure in TBM is the identification of correct templates, where the accuracy of template recognition and alignment essentially determines the accuracy of the final TBM models. In an effort to recognize structure templates that have similar folds to the query, a variety of threading methods with different alignment scores and searching schemes have been developed in order to match the query sequence and template structures [8,11–14]. The success of these methods is, however, often limited to the targets that have closely-homologous templates in the PDB. When the evolutionary relationship between the query and template is more distant, typically when the sequence identity is below 30% [15], the query-template alignment accuracy sharply declines. Thus, although there is evidence that the PDB library is complete and sufficient to solve the protein structure prediction problem [16], modeling of distantly-homologous proteins using TBM remains a major challenge in the field.

Recently, sequence-based contact prediction between residue pairs using co-evolution [17–20] and machine learning [21–25] has shown considerable promise for improving the modeling accuracy of distantly- and non-homologous proteins. The major advantage associated with contact-based modeling of protein structures is that correct contact maps, even those with a small fraction of coverage, can significantly reduce the conformational space needed to be searched during the folding simulations, while helping identify the global folds of proteins with complicated topologies. However, the contact map information cannot be directly used in threading, since the query-template alignments in most threading methods are built on dynamic programming or hidden Markov models that require single-body potentials, while contact map information is inherently two-body.

Several efforts have been previously made to incorporate pair-wise contacts into dynamic programming and subsequent threading alignments [12,13,26,27]. For instance, PROSPECT introduced a contact term, associated with a uniform background probability, into its scoring function. Due to the lack of specificity, however, the contact potential utilized in this method is fairly noisy and the improvement from contacts is limited. Additionally, the method uses a Divide-and-Conquer algorithm for the alignment searching procedure that is computationally expensive. Another early attempt is PROSPECTOR [12,26], which uses a “partly-thawed” approach that evaluates the contact potential based on the previous rounds of alignment iterations; the solutions obtained by this method are, however, not exact. In a recent effort, map_align [27] proposed an iterative double dynamic programming algorithm that aligns contact maps. Nevertheless, this program cannot provide exact solutions as the results rely on the initial estimation of the similarity matrix, which is not always optimal.

Since direct alignment of contact maps is challenging, several other attempts [28–33] have been made to solve the contact map alignment problem using the eigen-decomposition strategy. For example, inspired by a previous study by Galaktionov and Marshalland [34], EIGAs [28] performs eigen-decomposition of a protein’s contact map, with each residue of the protein assigned one of the eigenvalues which has the closest angle between the corresponding eigenvector and that residue. Here, the angle is related to the coordinates of the eigenvector. The contact map of two structures can then be aligned by dynamic programming, where the alignment score for aligning a pair of residues is the absolute difference between the two eigenvalues associated with the two residues. While the neglect of the eigenvectors by this method allows for fast structure alignment, it can lead to suboptimal alignment accuracy. Therefore, an extended version, EIGA [29], was proposed based on the spectral methodology that starts from the initial alignment output by EIGAs. The alignment is then iteratively refined by multiple rounds of dynamic programming. In each iteration, the protein pair is aligned based on the eigenvalue-weighted eigenvector elements assigned to the residues, and the alignment is then used to update the assignment of the residue’s eigenvalue-weighted eigenvector element. Although EIGA utilizes more information than EIGAs in order to obtain more accurate alignments, it heavily relies on the quality of the initial alignments generated by EIGAs. SABERTOOTH [30] is another protein structural alignment method based on the first principal eigenvector of the contact matrix. Since this method only uses one-dimension of the contact eigenvector, which may result in much of the information encoded in a contact map being lost, the alignment accuracy can be poor. Similarly, Al-Eigen [31] approximates contact maps by using the top eigenvectors generated by eigen-decomposition, and determines the similarity between two contact maps by global alignment of the principal eigenvectors. While all of these methods show promise for structure alignment guided by eigen-decomposed contact maps, they cannot be directly used for threading, where the native contact map of the query protein is unknown.

Recently, some methodologies have been extended specifically for threading. For instance, SABERTOOTH [32] was extended for sequence alignment, where the principal eigenvector of a protein is predicted by a neural-network whose input feature is the Position-Specific Scoring Matrix (PSSM) generated by PSI-BLAST [35], with the alignment approach identical to the former version of SABERTOOTH. More recently, EigenTHREADER [33] was developed to extend Al-Eigen to enable threading by predicting a protein’s contact map starting from its sequence, and then searching a library composed of contact maps for known structures. While these methods can perform threading based on predicted contact maps, they do not consider other linear features such as sequence profiles and secondary structure information, which can be used to further improve the alignment accuracy.

**Fig 1:**
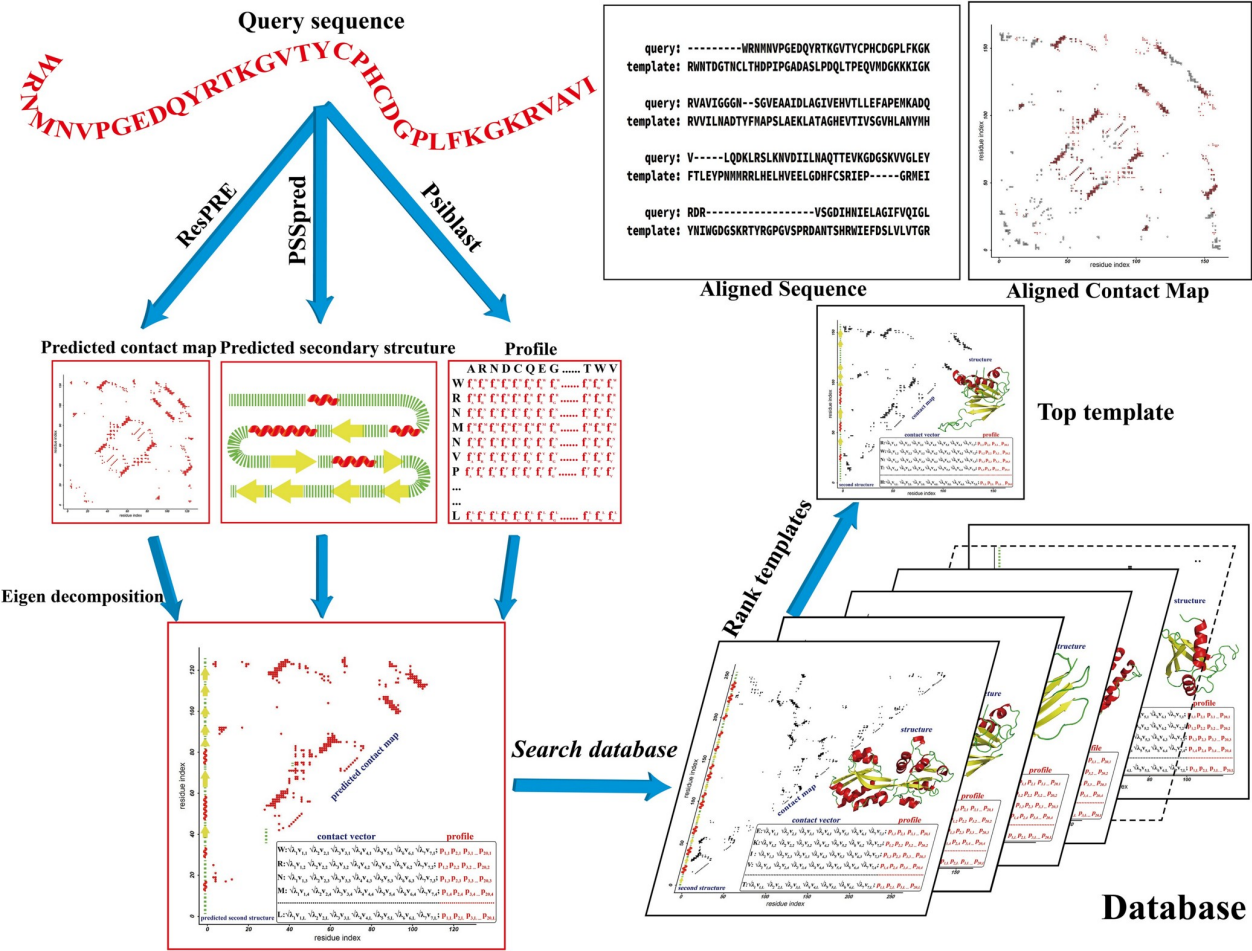
CEthreader flowchart. The pipeline consists of four major steps: contact map prediction, contact matrix eigen-decomposition, database searching and contact score-guided template selection.

In this study, we propose a new contact-based threading method, called CEthreader (Contact Eigenvector-based threader), which first creates contact map predictions using deep residual neural-network training. The contact map matrix is then represented by the cross product of single-body eigenvectors through the eigen-decomposition technique, following the idea of Al-Eigen [31]. Finally, extensive contact-based alignment searching, built on a newly developed dot-product scoring function used to align contact eigenvectors, is performed through dynamic programming. In addition to the contact maps, multiple inherent features, including secondary structure prediction and sequence profiles, are incorporated to further enhance the alignment accuracy (see the flowchart in **Fig 1**). Multiple large-scale benchmark tests were conducted to carefully examine the strengths, weaknesses and potential of the new contact-based threading approach. Comparison of CEthreader to start-of-the-art contact- and profile-based threading algorithms and pure contact- or template-guided folding approaches demonstrated the significant advantages associated with using our composite contact and profile-guided threading approach for distant-homology fold-recognition and modeling. The CEthreader online server and standalone package are freely available at https://zhanglab.ccmb.med.umich.edu/CEthreader.

## Results

### Construction of the template library and benchmark datasets

Two libraries of non-redundant protein structure templates were collected to test CEthreader. The first was taken from the I-TASSER threading template library [5], which consists of 51,376 single-domain structures with a sequence identity cutoff of 70% (see https://zhanglab.ccmb.med.umich.edu/library/). The second library was collected from SCOPe [36], which consists of 23,000 single-domain structures after filtering the redundant entries at the 95% sequence identity cutoff.

The threading methods were examined using two benchmark protein sets. The first (Set-I) consisted of 614 non-redundant protein domains taken from the PDB that satisfied the following criteria: (*i*) pair-wise sequence identity is <30% to any other selected protein in Set-I; (*ii*) each protein had at least one template with a TM-score >0.5 as identified by TM-align [37] structural alignment search through the I-TASSER template library [5]. Based on LOMETS [38], which categorizes proteins as Easy or Hard threading targets depending on the significance scores of multiple threading programs, this dataset contained 403 Easy targets (for which at least one program in LOMETS had a significant template hit) and 211 Hard targets (for which no program in LOMETS had a significant hit).

The second benchmark set (Set-II) was taken from the SCOPe database and constructed in two steps. First, we retrieved 5,497 structures from the SCOPe that satisfied the following requirements: (*i*) pair-wise sequence identity is <95% to any other selected protein in Set-II; (*ii*) structure came from a Fold group that contained at least two Superfamilies. Next, we randomly selected one representative target from each Superfamily, which resulted in 539 targets from 116 Fold groups. Based on LOMETS, this set contained 379 Easy and 160 Hard targets.

In both datasets, any protein that shared the same Superfamily with or had a sequence identity >30% to the CEthreader training proteins was excluded. In addition, proteins that had a sequence identity >40% to any protein in the ResPRE training set were excluded from both datasets. This slightly relaxed sequence identity cutoff to the ResPRE training set is mainly due to the fact that the ResPRE training set is large, including about 5,600 high-resolution protein structures in order to facilitate effective deep learning training, and the use of the same cutoff of 30% could result in an insufficient number of threadable structures in both benchmark datasets. For example, there were 614 protein targets in Benchmark Set-I when using a 40% sequence identity cutoff; however, using a 30% sequence identity cutoff would have reduced the number of targets in Benchmark Set-I to 239.

### Benchmark test on CEthreader

CEthreader was first tested on the 614 proteins in Benchmark Set-I, each of which was threaded through the I-TASSER template library, where any homologous templates with a sequence identity >30% to the query were excluded.

#### Effect of component scores on CEthreader alignment

**Fig 2:**
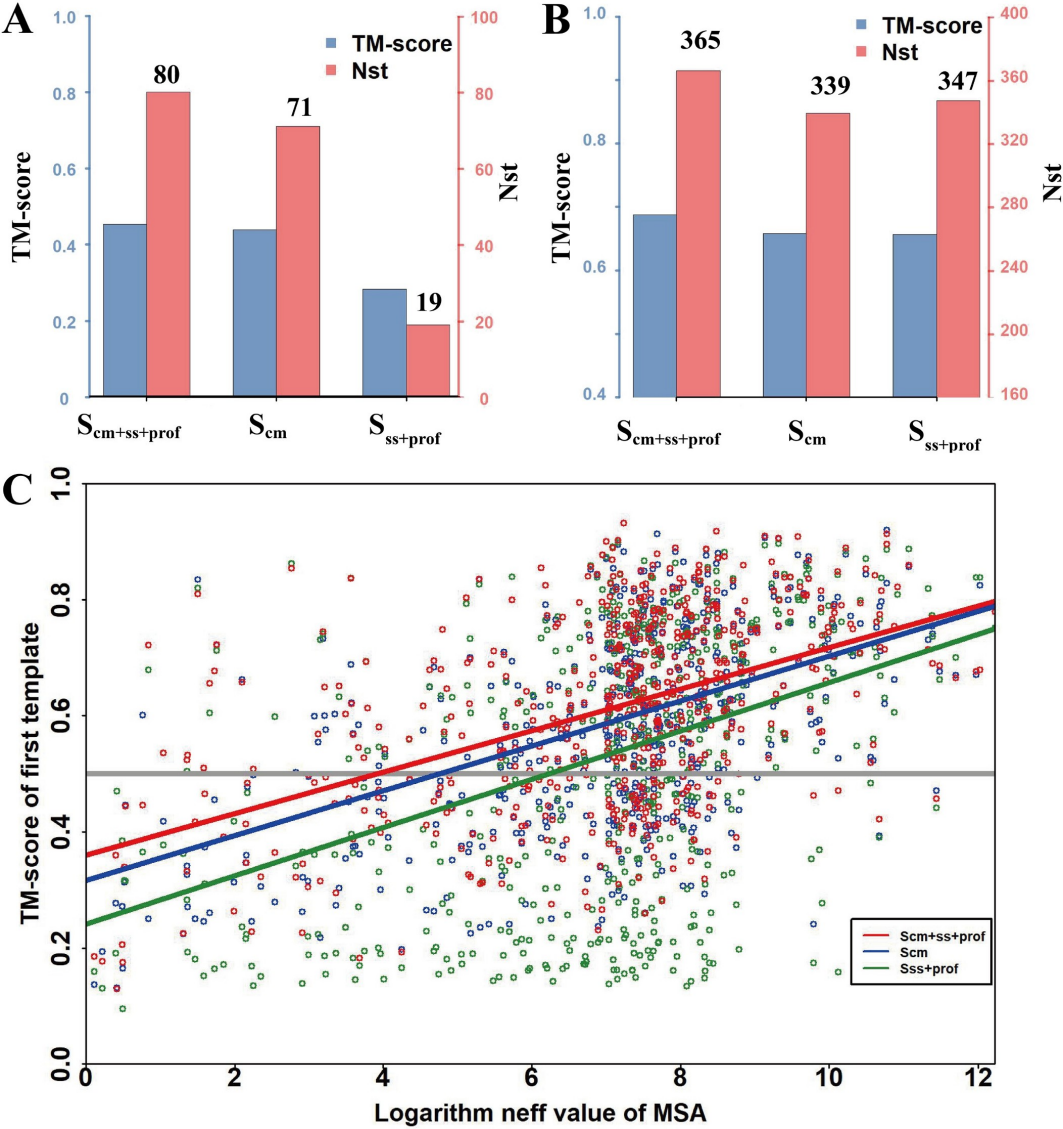
Performance of CEtheader on Benchmark Set-I, which was collected from the I-TASSER database. TM-score and *N*_*st*_ (number of targets with first template TM-scores >0.5) histograms for CEthreader using three different scoring functions on the 211 Hard targets (A) and 403 Easy targets (B). (C) The TM-score of the first template identified by CEthreader as a function of the logarithm of the MSA *Neff* value, where three scoring functions- *S*_*ss*+*prof*_ (green points), *S*_*cm*_ (blue points) and *S*_*cm+ss*+*prof*_ (red points)- were used by CEthreader. Linear regression was used to fit the correlation relationship between TM-score and MSA *Neff* value for the three scoring functions, where the fitted relationships for *S*_*ss*+*prof*_ (green line), *S*_*cm*_ (blue line) and *S*_*cm+ss*+*prof*_ (red line) are y = 0.242+0.042x, y = 0.317+0.039x, and y = 0.361+0.036x, respectively.

While sequence profile information derived from multiple sequence alignments (MSAs) for query and template proteins has been effectively used to detect homologous templates in many threading methods [8,14], it is often less effective for proteins that lack templates with closely-homologous sequences. Here, we explore the possibility of using contact map information (*S*_*cm*_ in **Eq 5**in **METHODS**) to enhance the ability to recognize distant-homology templates. To examine the impact of the different components of the CEthreader scoring function on the accuracy of template identification, we list in **Fig 2A** the average TM-scores of the first templates identified by using *S*_*cm*_ (contact map information only), *S*_*ss*+*prof*_ (profile and secondary structure information), or *S*_*cm*+*ss*+*prof*_ as the scoring function. First, the data show that contact-based threading using *S*_*cm*_ alone can identify better quality templates for Hard targets than traditional profile and secondary structure-based alignments, where the average first template TM-score for *S*_*cm*_ was 0.439 and 71 out of the 211 Hard targets had templates with TM-scores >0.5, as compared to an average first template TM-score of 0.284 and 19 targets with correctly identified templates for *S*_*ss*+*prof*_ (see more TM-score and RMSD comparisons in **S1 Table**). The poor performance by the latter method can be attributed to the fact that profile-profile alignments rely on the evolutionary relationship between the query and templates, which is much less accurate here as homologous templates have been excluded; meanwhile, the number of homologous sequences is low for the Hard targets (~227, on average) which further reduces the reliability of the profile-based alignments. On the other hand, ResPRE [39], a deep-learning-based contact map predictor (see **METHODS**), can generate contact predictions with reasonable accuracy even for targets with few homologous sequences, thus helping capture the global structural similarity between the query and templates in the absence of a reliable profile match.

When the *S*_*cm*+*ss*+*prof*_ score was used, the average TM-score of the first identified templates increased to 0.453, which was 3.2% (or 59.5%) higher than that obtained by contact-based threading using *S*_*cm*_ (or profile and secondary structure-based threading using *S*_*ss*+*prof*_), with a *p*-value of 2.3E-03 (or 7.8E-30) calculated using the Wilcoxon signed-rank test. Accordingly, the number of targets whose templates were correctly identified (TM-score >0.5) using *S*_*cm+ss*+*prof*_ was 80, which was higher than the number identified by either individual scoring function *S*_*cm*_ (71) or *S*_*ss*+*prof*_ (19). The data suggest that the combined scoring function helps to accurately align the query proteins to their templates and to identify correct templates more effectively than the scoring functions that were solely based on contact or profile and secondary structure information alone.

In **Fig 2B**, we also list the threading results for the 403 Easy targets that had homologous templates in the PDB. Although profile alignments have been proven to be highly efficient at detecting closely-homologous templates, the results show that contact map information can still help enhance the alignment accuracy for the selected Easy targets. This is demonstrated by the fact that the average TM-score of the first templates identified by CEthreader using *S*_*cm*+*ss*+*prof*_ (0.687) was about 4.7% higher than the corresponding templates detected using *S*_*ss*+*prof*_ (0.656) with a *p*-value of 1.1E-15, where the number of targets whose templates were correctly identified by the former (365) was also higher than the latter (347). When considering each of the 614 test targets together, the average TM-score of the first templates detected by *S*_*cm*+*ss*+*prof*_ (0.607) was 15% higher than the average TM-score of the first templates identified by *S*_*ss*+*prof*_ (0.528), which corresponds to a *p*-value of 2.1E-45; these data suggest that CEthreader can significantly improve the alignment accuracy and the number of correctly identified templates for both Hard and Easy targets.

Here, it is worthy of noting that the performance of our *S*_*ss*+*prof*_ score was slightly worse than that of the state-of-the-art secondary structure and profile-based method, HHsearch [8], for both Hard (TM-score = 0.284 vs TM-score = 0.314) and Easy targets (TM-score = 0.656 vs TM-score = 0.682, see more comparisons with other programs in **S2 Table**, which will be discussed later, where our *S*_*ss*+*prof*_ score is called PPA). Even though the non-contact-based components (secondary structure and profile matching) do not produce as accurate results as HHsearch, the performance of the full CEthreader algorithm, which couples the non-contact-based components (secondary structure and profile matching) with the contact-based component (contact eigenvector matching), is significantly better than all other methods, underscoring the importance of contact information to the CEthreader method (**S2 Table**).

In **Fig 2C**, we further examined the effective number of homologous sequences, *Neff* (defined in **Eq. S1** in **S1 Text**), which is required for each scoring function to identify good templates with TM-scores >0.5. The figure lists the TM-scores of the first templates identified by CEthreader using different scoring functions (*S*_*ss*+*prof*_, *S*_*cm*_ and *S*_*cm*+*ss*+*prof*_) versus the logarithm of the MSA *Neff* values. Despite the large variations, we use linear regression to fit the correlation relationship between the template TM-score and the logarithm of the *Neff* value for the three scoring functions. The intersection points between the fitted lines and the TM-score = 0.5 horizontal line can be considered as an approximate cutoff to determine what *Neff* values are required for each scoring function to identify good templates. It was found that threading with the *S*_*ss*+*prof*_ scoring function has the highest dependency on the MSA *Neff* value with a cutoff of 2^6.14^ (≈64), which means that on average the *S*_*ss*+*prof*_ scoring function may detect good templates only when the *Neff* is >64. On the other hand, the *S*_*cm*_ scoring function, which only uses contact information to guide the threading alignments, has a relaxed *Neff* cutoff of 2^4.69^. The combination of both scores, *S*_*cm*+*ss*+*prof*_, has the widest applicability since it can identify good templates for targets with the lowest *Neff* values (only >2^3.86^). These data suggest that CEthreader has the ability to identify good templates for targets whose MSAs have relatively few effective sequences, thereby extending the applicability and usefulness of CEthreader, especially for Hard targets.

#### Why does the combination of contacts with profile and secondary structure work?

**Fig 3:**
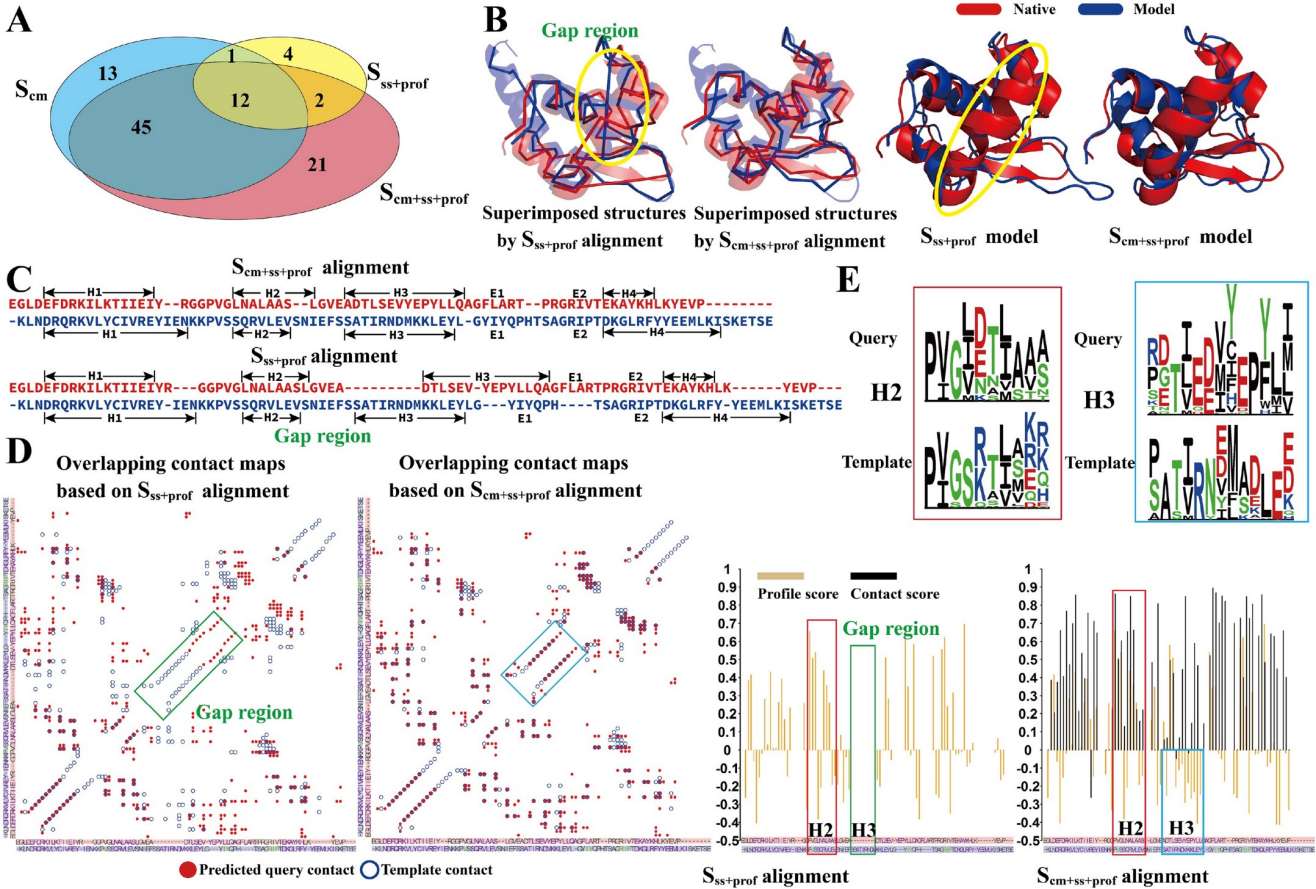
Performance of CEtheader on the 211 Hard targets in Benchmark Set-I from the I-TASSER database. (A) Venn diagram for the number of targets with first template TM-scores >0.5 using different scoring functions. (B) Overlay of the CEthreader template (left panel) and MODELLER models (right panel) on the native structure from the RuvB Holliday junction branch migration motor (PDB ID: 1in4A1), where two scoring functions- *S*_*ss*+*prof*_ and *S*_*cm*+*ss*+*prof*_ - were used by CEthreader. (C) Query-template alignments using different scoring functions for 1in4A1, where H1-4 and E1-2 are secondary structure segments assigned from the native PDB structure. (D) Overlay of the query (open circles) and template (closed circles) contact maps obtained using *S*_*ss*+*prof*_ (left panel) or *S*_*cm*+*ss*+*prof*_ (right panel), where helix, strand and loop regions are marked with purple, green and grey characters along the sequences. (E) Contribution of profile- (yellow) and contact-based (black) scores to the CEthreader alignments when using *S*_*ss*+*prof*_ (left) or *S*_*cm*+*ss*+*prof*_ (right).

To examine the effectiveness and rational of the combined scoring function for fold-recognition, we present in **Fig 3A** a Venn diagram of fold-recognition results using the three scoring functions, *S*_*cm*_, *S*_*ss*+*prof*_ and *S*_*cm*+*ss*+*prof*_, on the selected Hard targets. We found that the 80 targets whose templates were correctly detected by *S*_*cm*+*ss*+*prof*_ with a TM-score >0.5 were not a simple overlap of the 71 targets found using *S*_*cm*_ and the 19 targets found using *S*_*ss*+*prof*_. Instead, the composite scoring function helped detect correct templates for 21 new targets that were not identified using either contact or profile and secondary structure-based threading search alone.

In **Fig 3B** (Left panel), we present a representative example from the RuvB Holliday junction branch migration motor (PDB ID: 1in4A1), for which the templates identified using *S*_*ss*+*prof*_ and *S*_*cm*+*ss*+*prof*_ are superimposed onto the native structure separately. The match based on profile and secondary structure resulted in an incorrect alignment shift starting from the third helix region (H3), where the resulting alignment gap is shown in **Fig 3C** and highlighted with a circle in **Fig 3B**. Under the pressure of enforcing contact map matching between the query and template, however, the correct alignment was restored when using *S*_*cm*+*ss*+*prof*_, where each of the four helix regions of the template was correctly aligned to the query. This improvement can be clearly seen in **Fig 3D**, where the gap region corresponds to the mis-match between the query and template contact maps (Left panel), which is removed after considering the contact match score (*S*_*cm*_) (Right panel). As a result, the TM-score of the template identified using *S*_*ss*+*prof*_ was 0.46, which was considerably lower than that by *S*_*cm*+*ss*+*prof*_ (0.61). This example highlights the usefulness of coupling contact maps with profile and secondary structure information to improve the alignment accuracy even when the same template is identified. The Right panel of **Fig 3B** displays the superposition of the native structure with the full-length models constructed by MODELLER [4] based on the template alignments, where the model built from the *S*_*ss*+*prof*_ template alignment (with a model TM-score = 0.53) contains a long loop between two strands (E1 and E2) due to the alignment shift at H3. On the other hand, the use of *S*_*cm*+*ss*+*prof*_ leads to a better full-length model along each of the secondary structure regions with an improved TM-score (= 0.68).

In **Fig 3E**, we quantitatively analyze the contribution of profile and contact information to the query-template alignment for 1in4A1. We observed that the profile score was positive in the H1 and H2 regions for both the query and template due to the conservation of amino-acid composition, as evident from their profiles (see the top left panel of **Fig 3E**). As a result, the profile match is dominant when aligning the H1 and H2 regions of the query and template, and hence contact information does not have a significant effect on the alignment of these regions. However, the profile score is highly negative when the H3 region of the query is aligned to that of the template due to their different amino acid compositions in this region (see top-right panel of **Fig 3E**). Hence, the dynamic programming algorithm tends to add gaps in this region to maximize the final score of the profile-guided alignment. On the other hand, the contact information is highly conserved in the H3 region (positive score in the aligned H3 region), and thus the net score in this region has a positive contribution to the final score. Nevertheless, it is noteworthy that the overall profile information is reliable for this example, since approximately 1,000 homologous sequences were obtained for both the query and template sequences using PSI-BLAST [35] with an *E*-value cutoff of 0.01. This particular example highlights the importance of profile information when aligning regions where the amino acid composition is conserved, while also showing how contact map prediction can help fix the alignment errors along the regions where the profiles for the query and template do not match.

Furthermore, we conducted an analysis to examine whether and how the performance of CEthreader varies based on the relative difficulty of identifying templates for the Hard targets, where the latter is quantitatively assessed by the structural similarity between the target and the best possible template identified by TM-align [37] from the PDB. As shown in **S1 Fig**, we found that as the TM-score between the target and the best template increased, the performance of CEthreader-detected templates (with both the best structural alignment and the CEthreader alignments) generally increases, meaning that the performance of CEthreader does have a weak but positive correlation with the similarity between the target and templates. Nevertheless, the TM-scores of CEthreader-identified templates were uniformly around 0.14 units lower than those of the best possible templates for the 211 Hard targets. When comparing the alignment results on the same template, the alignment quality of CEthreader, in terms of TM-score, was around 0.06 units lower than that produced by the structure alignment program, TM-align. These data show that there are considerable room for CEthreader improvement in both template detection and alignment construction.

#### Reduction of time complexity using a hybrid threading approach

We use eigen-decomposition to convert the two-body contact map potential into a single-body potential, where the accuracy of the contact matrix representation increases with an increased number of eigenvectors (see **METHODS**). However, the time cost also increases rapidly when more eigenvectors are taken into account when aligning the query-template contacts, because a total of 2^*K*^ possible alignments must be enumerated when considering *K* eigenvectors so that the sign of every involved eigenvector can be decided. This is particularly an issue when threading through a large size database. For example, when we set *K* = 7, the average time required to thread a query sequence of 200 residues through our database of 51,376 templates is ~130 hours. In order to reduce the time cost while maintaining the alignment accuracy, we implement a hybrid threading approach that combines both greedy and enumerative searching strategies.

First, we use a greedy searching strategy to scan the whole database with a small *K* (= 2) and rank all the templates based on *CMOq = O*(*CM*^*Q*^, *CM*^*T*^)/*N*(*CM*^*Q*^), where *N*(*CM*^*Q*^) is the number of contacts used by CEthreader and O(*CM*^*Q*^,*CM*^*T*^) is the number of overlapped contacts between the aligned regions of the query and template proteins. For each query-template pair, this greedy strategy attempts to invert the signs of each eigenvector in turn, starting with the eigenvector associated with the largest eigenvalue. Once a sign inversion is set, this process is performed for the rest of the eigenvectors until all *K* signs are set. This greedy algorithm performs only 2^*K*^ alignments, which significantly reduces the time complexity when compared to the enumerative searching strategy where all 2^*K*^ alignments must be performed. Next, based on the ranking results in the first step, we select the top 1,000 templates for the query sequence and re-rank these templates using an enumerative searching strategy with a cumulative total of 7 eigenvectors. Although 254 (2^1^+2^2^+2^3^+2^4^+2^5^+2^6^+2^7^ = 254) alignments are performed for each template, the number of templates is significantly reduced, which means all of the query-template alignments can be conducted with a reasonable cost and an average CPU time of ~2 hours.

**S2 Fig** in the SI shows the performance of CEthreader on the 211 Hard targets using different searching strategies and different numbers of eigenvectors. When only one eigenvector was used in the first step, the greedy searching strategy could detect correct templates for 57 targets, and the average time cost was ~1.4 hours. After further optimization based on the enumerative searching of the top 1,000 templates, the number of correct templates increased to 77. When two eigenvectors were used in the first step, correct templates for 68 targets could be identified by the greedy searching algorithm with an average time cost of ~2.4 hours, and correct templates were recognized for 79 targets in the second step of searching. Although the performance based on greedy searching increased with an increase in the number of eigenvectors, the final performance based on the hybrid searching strategy did not change much. In fact, the number of correct templates identified after implementing our greedy heuristic was very close to the number found after ~130 hours of enumerative searching through the whole template library of 51,376 proteins based on a cumulative total of 7 eigenvectors. Therefore, the hybrid searching strategy with only 2 eigenvectors used during the greedy searching step achieves reliable performance but with significantly reduced time complexity.

### Comparison of CEthreader to other state-of-the-art threading approaches

To further examine the impact of contact map information on threading, we compare the performance of CEthreader to other state-of-the-art profile- and contact-based threading programs. The comparisons were made on our two benchmark sets (Set-I and Set-II), where each target from Set-I and Set-II were searched through either the I-TASSER template database or the SCOPe database, respectively.

#### Test on Benchmark Set-I: Ability to recognize the most accurate templates

**Table 1**shows the CEthreader threading results in comparison to eight state-of-the-art threading programs. The results were obtained by threading the 211 Hard targets in Set-I through the I-TASSER template library, where all templates that had a sequence identity >30% to the query sequence were excluded. The data show that the average TM-score of the identified templates by CEthreader was 0.453, which was at least 9% or 45% higher than the average TM-scores by other contact-based methods or profile-based methods, respectively. The contact-based methods included: map_align (0.414) and EigenThreader (0.413), while the profile-based methods included: HHsearch (0.313) and MUSTER (0.304) [14]. The TM-score comparisons between CEthreader and the other methods were also statistically significant as per the Wilcoxon signed-rank tests which had *p*-values <4.4E-06 for all of the comparisons. The table also shows that CEthreader could identify correct templates for 80 out of the 211 Hard targets, which was 1.35 times higher than the next best contact-based methods and 2.76 times higher than the next best profile-based method. The average RMSD by CEthreader was also lower than that of other programs, including the ones (HHsearch, SAM-T99 [40] and FFAS [41]) with significantly lower alignment coverage, which resulted in the higher TM-score.

**Table 1:** Performance of different threading methods on the 211 Hard targets from Benchmark Set-I. *P*-values were calculated between the CEthreader alignment TM-scores and other methods’ TM-scores using one-sided Wilcoxon signed-rank tests. Coverage is equal to the number of aligned residues divided by the length of the query sequence. *N*_*st*_ represents the number of targets with an identified template whose TM-score was >0.5.

Methods	TM-score	*p*-value	RMSD (Å)	Coverage	*N*_*st*_
CEthreader	0.453	-	9.53	0.875	80
map_align	0.414	4.39E-06	11.48	0.896	59
EigenThreader	0.413	1.31E-09	10.15	0.850	49
HHsearch	0.313	2.13E-24	10.92	0.654	29
MUSTER	0.304	5.02E-28	13.94	0.869	23
PPA	0.284	7.75E-30	14.78	0.832	19
PROSPECT2	0.261	2.52E-35	16.63	0.915	10
SAM-T99	0.208	1.04E-34	11.17	0.528	10
FFAS	0.189	1.25E-35	14.39	0.674	7

The elevated performance of CEthreader compared to other state-of-the-art methods can again be attributed to the combination of the complementary structure (contact maps) and profile information, while other excellent profile-based programs, such as HHsearch and MUSTER, use profile and single-body local structural information (such as secondary structure), which are not sufficient to detect correct templates for many Hard targets. More importantly, by carefully coupling the profile information, CEthreader performs significantly better than other contact-based methods, such as map_align and EigenThreader, which rely solely on contact information to guide threading.

Furthermore, although CEthreader was primarily developed for threading Hard targets, the alignments compare favorably with other profile-based threading programs for the 403 Easy targets, with a *p*-value <2.2E-03 relative to the best of the profile-based programs and <3.2E-23 to the best of the contact-based programs (see **S2 Table**). Note that without the use of profile information, other contact-based methods, such as map_align and EigenThreader, perform worse than profile-based methods (HHsearch, MUSTER, and PPA) on the Easy targets. The head-to-head comparisons between CEthreader and EigenThreader or map_align can be found in **S3 Fig**.

As discussed above, we have used a sequence identity cutoff 40% (instead of the <30% cutoff enforced between Set-I and the CEthreader training set) to filter proteins in Benchmark Set-I homologous to the ResPRE training dataset. In order to justify the reasonability of selecting the 40% sequence identity cutoff, we divided Benchmark Set-I with its 614 targets into two subsets: the first subset includes 239 targets with sequence identities <30% to the ResPRE training set, and the second contains 375 targets with sequence identities between 30% and 40% to the ResPRE training set. **S3 Table** shows that CEthreader is still the best performing threading method compared to other profile- and contact-based methods for both of the subsets, which is consistent with the results for the entire Benchmark Set-I as shown in **Tables 1**and **S2**, even though the results for the subset with sequence identities *≥*30% are slightly better than those for the subset with sequence identities <30%.

#### Test on Benchmark Set-II: Ability to recognize folds in different Superfamilies

Despite filtering out templates with high sequence identities to the target proteins (>30%), templates identified in Benchmark Set-I may still have evolutionary relationships to the target proteins. Meanwhile, the distribution of the evolutionary distances between the query sequences and templates is highly uneven and varies from case-to-case. To conduct a more rigorous quantitative assessment of the ability of each threading program to recognize different levels of distantly-homologous templates, we performed a second test using Benchmark Set-II, which was collected from the SCOPe database. SCOPe is organized using a stringent hierarchy of protein structures, ranging from Class, Fold, Superfamily to Family, with members in a lower level of hierarchy having a closer evolutionary and structural similarity.

**Table 2**summarizes the results for five state-of-the-art threading programs for which all templates within the same Superfamily as the query were excluded. The average TM-score of the first templates identified by CEthreader from the SCOPe database was 0.483, which was significantly higher than that by the next best contact-based method, i.e., map_align (0.464), or profile-based method, i.e., HHsearch (0.301), with Wilcoxon signed-rank *p*-values <5.0E-05. Out of the 539 targets, CEthreader detected templates with the same Fold as the query in 323 cases, resulting in a SCOPe Fold-recognition success rate of 59.9%; this is much higher than the success rate of EigenThreader (50.3%) or HHsearch (28%). These data show that CEthreader has a stronger ability to detect templates with similar Folds even in the absence of a Superfamily-level homologous relationship to the query. In the lower panel of **Table 2**, the query proteins are categorized as Easy (379 targets) or Hard (160 targets) based on their LOMETS classification, where similar trends in TM-score and success rate are observed for both Easy and Hard targets.

**Table 2:** Threading results obtained by different methods for the 539 proteins from Benchmark Set-II. *P*-values were calculated between the CEthreader alignment TM-scores and other methods’ TM-scores using one-sided Wilcoxon signed-rank tests; *N*_*ST*_ represents the number of targets with a first identified template whose TM-score was >0.5; the success rate is equal to the fraction of targets whose Folds were correctly recognized, i.e., the first identified template had the same Fold as the query.

	Methods	TM-score	*p*-value	*N*_*ST*_	Success rate
All (539)	CEthreader	0.483	-	323	59.9%
	map_align	0.464	4.99E-05	261	48.4%
	EigenThreader	0.450	1.31E-21	271	50.3%
	HHsearch	0.301	2.84E-79	151	28.0%
	MUSTER	0.285	6.57E-85	99	18.4%
Easy (379)	CEthreader	0.493	-	228	60.2%
	map_align	0.478	5.62E-03	195	51.5%
	EigenThreader	0.462	6.43E-16	199	52.5%
	HHsearch	0.326	1.18E-55	119	31.4%
	MUSTER	0.304	2.68E-61	80	21.1%
Hard (160)	CEthreader	0.459	-	95	59.4%
	map_align	0.431	7.65E-04	66	41.3%
	EigenThreader	0.422	2.26E-07	72	45.0%
	HHsearch	0.242	1.65E-25	32	20.0%
	MUSTER	0.241	8.37E-26	19	11.9%

**Fig 4:**
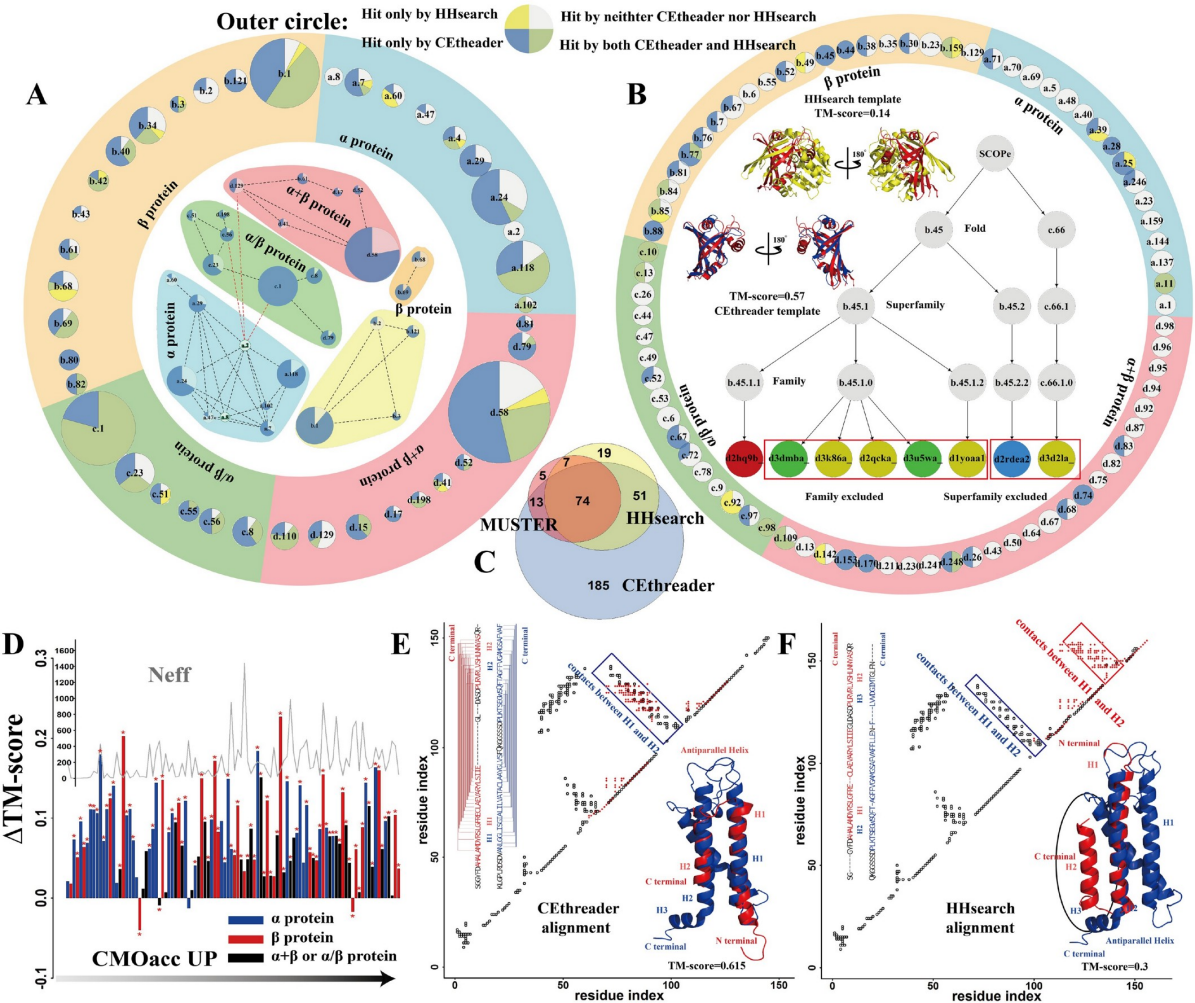
Performance of CEthreader and HHsearch on Benchmark Set-II, which was collected from the SCOPe database. (A) The ability of CEthreader and HHsearch to recognize Folds from 39 large Fold groups with ≥4 Superfamilies. The size of a pie chart is proportional to the number of Superfamilies in the Fold group. The area of the sectors of each pie chart in the Outer circle is proportional to the number of correct hits by each different method, while that in the Inner circle represents the portion of Superfamilies hit (blue) or not hit (white) by CEtheader. A dotted line connects two Folds if the average TM-score between them is >0.4. (B) Same as (A) but for 77 small Fold groups with 2 or 3 Superfamilies. The Inner disc shows a representative example from d2hq9b_ as indicated by the red circle in the SCOPe tree, where the first templates by CEthreader (blue) and HHsearch (yellow) are superimposed on the native (red) separately. (C) Venn diagram for the number of targets with the correct Fold detected by CEthreader, HHsearch, and MUSTER on all 539 Benchmark Set-II proteins. (D) TM-score difference between CEthreader and HHsearch (ΔTM-score) versus ResPRE contact prediction accuracy (CMOacc) and the number of effective sequences in the MSAs (*Neff*) for 116 Hard targets from (A) and (B). Stars indicate that the TM-score difference between CEthreader and HHsearch is statistically significant as determined by Wilcoxon signed-rank tests. (E, F) Overlay of the predicted contact map and the contact map of the templates identified by CEthreader or HHsearch for the example from d2db7b_. The lower right corner shows the structure superposition of the templates (blue) and the native (red), while the upper left corner shows the query-template alignments with connected lines marking contacts between two residues.

**Fig 4**shows a more detailed illustration of the ability of CEthreader and HHsearch to detect non-homologous templates from Fold groups of different sizes. In the outer circle of **Fig 4A**, there are 39 Fold groups with each containing at least 4 Superfamilies, where 29 groups were threadable by both CEthreader and HHsearch, 8 other groups were threadable only by CEthreader, and none of the groups were threadable by HHsearch but not by CEthreader. Here, "threadable" refers to cases for which the identified template and query proteins share the same Fold. For the 29 cases, both CEthreader and HHsearch demonstrated good performance at recognizing Folds with a high number of Superfamilies. On the other hand, recognition of Folds became more challenging when the number of Superfamilies in a Fold group was smaller, as statistically there was a lower chance of recognizing the correct template. In **Fig 4B**, we list 77 Fold groups in the SCOPe set that had ≤ 3 Superfamilies, where 39 of them could not be detected by either CEthreader or HHsearch. There were, however, 26 Fold groups where CEthreader identified a higher number of correct templates (with the same Fold) than HHsearch, while HHsearch did so only in 5 Fold groups. Overall, as shown in **S4 Table** CEthreader detected correct templates in the same Fold group as the query in 72 cases, while HHsearch did so in 44 cases out of the 116 Fold groups, which were selected after excluding the Superfamily-level homologs.

In the inner disc of **Fig 4A**, we further analyze the evolutionary connection between different Folds in the SCOPe database in order to investigate what types of Folds are recognizable by CEthreader. For this purpose, a dotted edge is drawn between two Folds if the average TM-score between the Folds was >0.4, where Folds were clustered based on the edges using Walktrap [42] (**S5 Table**). Here, the TM-score cutoff of 0.4 was chosen as multiple Fold groups could be formed with no singleton cluster at this cutoff. For the 39 Folds with ≥4 Superfamilies, the four clusters approximately corresponded to the four Classes of Folds, i.e., α-, β-, α+β, and α/β proteins. The figure shows that CEthreader tends to have better performance for Folds of smaller degree (less edges) and/or larger size (more Superfamilies), especially for those in large clusters. For example, in the blue region of the inner disc that corresponds to the α-protein Class, CEthreader failed to detect Folds a.2 and a.8 that had both large degree and small size, while it showed excellent performance in identifying Folds a.24 and a.118, both of which were of small degree and large size, and Fold a.102, which was of small degree. Since Folds with larger sizes comprise more Superfamilies, it is easier for CEtheader to detect a correct Fold. Additionally, Folds with smaller degree have fewer neighbors that are structurally similar to the Fold, thus there is a lower chance of mis-identifying the templates from other Folds by CEthreader, which utilizes structural information to identify templates.

In order to better illustrate the enhanced ability of CEthreader compared to HHsearch to recognize correct Folds, we present in the inner disc of **Fig 4B** an example from a fad-binding protein (SCOPe ID: d2hq9b_). When all templates with the same SCOPe Family to the query were excluded, both CEthreader and HHsearch could detect templates from the correct Fold group as highlighted by the yellow (for HHsearch) and green (for both CEthreader and HHsearch) circles in **Fig 4B**. When the templates with the same Superfamily to the query were excluded, however, CEthreader could still detect the top-ranked template (SCOPe ID: d2rdea2) with the same Fold (b.45) and a TM-score of 0.57 to the query, but HHsearch’s top-ranked template (SCOPe ID: d3d2la_) was from a different Fold (c.66) that had a TM-score of 0.14 to the query. This example illustrates the enhanced ability of CEthreader to detect distantly-homologous templates.

As a summary, we present in **Fig 4C** a Venn diagram for the number of cases for which the correct Fold was detected by CEthreader, HHsearch and MUSTER for all 539 targets in Set-II. It shows that the overlap between HHsearch and MUSTER was high, where 88% of targets whose Fold were correctly identified by MUSTER were also detected by HHsearch, probably due to the fact that both methods are built on the same principle of profile-profile alignment. On the other hand, due to the utilization of contact maps, CEthreader had a relatively lower overlap with HHsearch and MUSTER, where there were 185 cases (57%) whose Folds were correctly identified by CEthreader but not by HHsearch or MUSTER. Since CEthreader also adopts profile alignment information, most of the Folds successfully recognized by MUSTER and HHsearch were also recognized by CEthreader, which again demonstrates the advantages of using the composite profile- and contact map-based scoring function for threading alignment.

#### Test on Benchmark Set-II: Impact of contact map accuracy on Fold-recognition

To specifically examine the potential of contact-based threading, we collected a subset of 102 modelable proteins from the 160 Hard targets in Benchmark Set-II, each of which had at least one template with TM-score >0.5 as identified by searching the SCOPe library using TM-align. In total, TM-align identified 15,440 templates with TM-scores >0.5 for the 102 selected proteins, with around 150 templates for each query protein. **Fig 4D** shows the difference in average TM-scores between CEthreader and HHsearch query-template alignments (ΔTM-score) for the 102 proteins, as a function of contact prediction accuracy, *CMOacc* = *O*(*CM*^*pred*^,*CM*^*native*^)/*N*(*CM*^*native*^), where *N*(*CM*^*native*^) is the number of contacts in the native structure and *O*(*CM*^*pred*^,*CM*^*native*^) is the number of overlapped contacts between the native and the top *N*(*CM*^*native*^) predicted contacts.

The data show a modest but obvious correlation between the improvement of CEthreader over HHsearch and the *CMOacc* values. For example, the average *Δ*TM-score = 0.087 for the targets with *CMOacc* >0.5, while *Δ*TM-score = 0.073 for the targets with *CMOacc* below 0.5. There was also a correlation between *Δ*TM-score and the effective number of homologous sequences, *Neff*, where *Δ*TM-score = 0.092 (or 0.081) for targets with Neff > 128 (or < 128). This is understandable because the contact prediction in ResPRE was based on training using multiple sequence alignments [43], where a higher *Neff* corresponds to a more complete sequence profile and therefore can result in, on average, better contact map prediction accuracy. Overall, *Δ*TM-score was >0 for 98 and <0 for 4 out of the 102 Hard targets. In 83 cases, the TM-score by CEthreader was significantly higher (with *p*-values < 0.05) than that by HHsearch, while HHsearch only had a significantly higher TM-score in 3 cases (see stars marked in **Fig 4D**). Here, the *p*-values were calculated using Wilcoxon signed-rank tests for all of the ~150 templates for each target.

In **Fig 4E** and **4F**, we present an example from the hypothetical protein MS0332 (SCOPe ID: d2db7b_) to illustrate the impact of contact map prediction on threading results. For this target, both CEthreader and HHsearch identified the Cyanobacterial Photosystem I protein (SCOPe ID: d1jb0l_) as the top template. The query had a four-helix bundle fold, but HHsearch could only align the C-terminal helix of the query (helix H2) to the C-terminal helix of the template (helix H3) based on profile and secondary structure information, which resulted in a low TM-score (= 0.3) compared to the native. On the other hand, utilizing the contacts predicted by ResPRE between the residues in helices H1 and H2, CEthreader correctly aligned all four helices between the query and template by maximizing the overlap between the contact maps of the query and template structures, which resulted in a query-template alignment with a TM-score of 0.615. This example again demonstrates how the predicted contact map information helps correctly align two proteins that do not share any homologous relationship.

### CEthreader goes beyond simple contact map prediction

The core of the CEthreader development involves the integration of contact maps with profile and secondary structure information to improve the threading alignment accuracy. Based on our former benchmark tests, the combination of contact maps with the profile and secondary structure features can help CEthreader to generate significantly better template alignments than the methods built on either profile or contact map alignment. To further examine their impact on the full-length homologous model constructions, **S6 Table** listed a summary of the TM-score and RMSD of the full-length models constructed by MODELLER based on the template alignments generated by CEthreader and other profile- and contact-based threading algorithms for the proteins in Benchmark Set-I. It was observed that the TM-scores of the first models generated by CEthreader/MODELLER are 0.708 and 0.476 for Easy and Hard targets, respectively, which were significantly higher than the corresponding average TM-scores for models built by EigenThreader/MODELLER (0.656 and 0.449) or HHsearch/MODELLER (0.698 and 0.330), with *p*-values <4.42E-05 for all cases. These data suggest that CEthreader helps improve the template alignments and full-length model construction for both Easy and Hard targets.

To further improve the modeling quality, we coupled the CEthreader threading alignments with the predicted contact map information using a recently developed contact-guided structural assembly pipeline, C-I-TASSER [44], to build full-length structural models. The C-I-TASSER folding engine is based on the classical template-based modeling approach, I-TASSER [5], but with a new energy term to account for the contact-map restraints from the deep neural network predictions (see **METHODS**). The CEthreader/C-I-TASSER folding engine was modified here to use the top five ranked CEthreader templates and the ResPRE contacts as restraints. In **S7 Table,** we presented a comparison between the full-length models constructed by the C-I-TASSER and the models generated by the pure template-based modeling tool, I-TASSER, starting from the same top five CEthreader templates for all 614 targets in Benchmark Set-I. As a control, we also listed the results from the models built by the Crystallography & NMR System (CNS) program [45], utilizing the same set of predicted contacts from ResPRE. For the 211 Hard and 403 Easy targets, the TM-scores of the first full-length models generated by the pure template-based structural assembly approach, CEthreader/I-TASSER, were 20.2% and 39.2% higher than that by ResPRE/CNS, respectively; which partly reflects the advantage of model construction using contact-guided threading templates over that using only predicted contact maps. When using CEthreader/C-I-TASSER, the TM-scores of the first models were further increased by 1.8% (41.7%) and 13.5% (36.5%) for Easy and Hard targets, respectively, compared to that by the CEthreader/I-TASSER (ResPRE/CNS) pipelines. The result illustrates how the synergistic interplay between contact-guided threading and contact-map predictions can overcome the weaknesses of both pure threading-based modeling and pure contact-based modeling methods.

**Fig 5:**
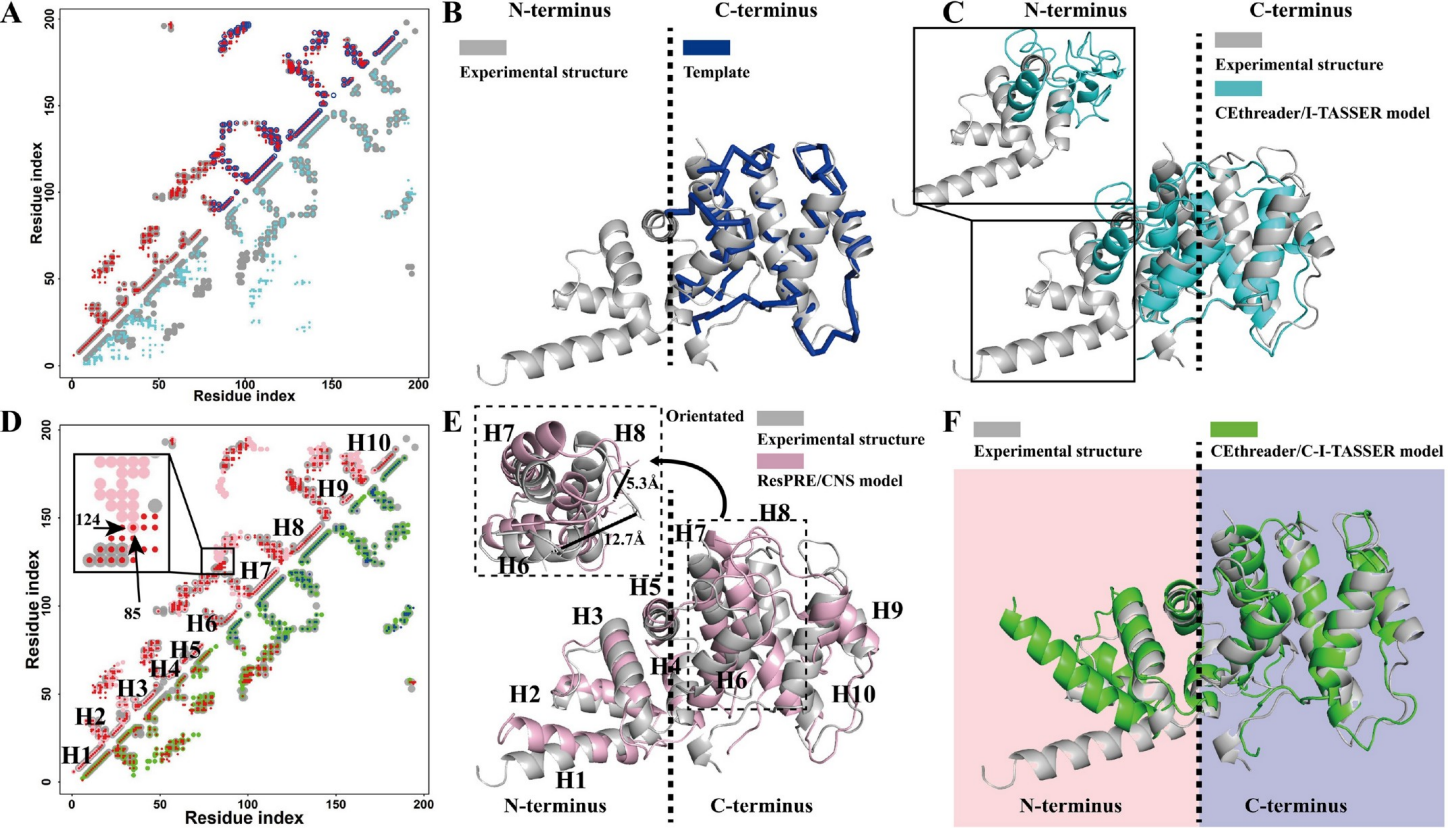
Modeling results for the ARF guanine-nucleotide exchange factor 1 (PDB ID: 1re0B). (A) Overlay of contact maps for the native query (gray circles), ResPRE-predicted contacts (red circles), CEthreader detected template (blue circles), and CEthreader/I-TASSER model (cyan circles). (B) The structure superposition of the templates (blue) and the native (gray). (C) The structure superposition of the CEthreader/I-TASSER model (cyan) and the native (gray). (D) Overlay of contact maps for the native query (gray circles), ResPRE-predicted contacts (red circles), overlapped contacts between the ResPRE prediction and the CEthreader detected template (blue circles), the ResPRE/CNS model (pink circles), and the CEthreader/C-I-TASSER model (green circles). (E) The structure superposition of the ResPRE/CNS model (pink) and the native (gray). (F) The structure superposition of the CEthreader/C-I-TASSER model (green) and the native (gray).

In **Fig 5**, we presented the modeling results for the ARF guanine-nucleotide exchange factor 1 (PDB ID: 1re0B), which is an α-protein consisting of 10 helices. For this target, ResPRE accurately predicted the contact map with a *CMOacc* of 0.915 (**Fig 5A**). Using the predicted contacts, CEthreader identified a template, 1xszA3 (**Fig 5B**), that fully covered the C-terminus of the target with a local TM-score of 0.808 (normalized by the length of the template) and a *CMOq* of 0.335, but had the N-terminal region completed missed. Despite the fact that the local alignment quality of the template was good, due to the low alignment coverage (59%), the full-length model generated by CEthreader/I-TASSER had a near random coil conformation in the N-terminus region, resulting in a relatively low overall TM-score of 0.578 (**Fig 5C**). These data show that template-based modeling approaches lose much of their accuracy in gap regions where the aligned template information is absent. On the other hand, since the predicted contact map overlapped well with the native contact map for the entire protein (**Fig 5D**), ResPRE/CNS constructed a full-length model with a slightly improved TM-score of 0.637 (**Fig 5E**). However, ResPRE/CNS utilized restraints from all of the predicted contacts (95% true positive contacts and 93% false positive contacts), where the false positive contact between residue 85 and 124 (black box in **Fig 5D**) drew helix H6 close to helix H8 in the C-terminus (**Fig 5E sub-figure**) during the CNS simulation. This caused the position to shift away from that found in the experimental structure and produced even more false positive contacts in the C-terminus of the final CNS model. Therefore, when only considering the C-terminus region, the TM-score of the ResPRE/CNS model was much lower than that of the CEthreader/I-TASSER model (0.477 vs 0.833). This analysis shows that pure contact-based modeling is still not as accurate as template-based modeling when high quality templates can be identified. In the CEthreader/C-I-TASSER pipeline, since both contact-map and threading template were considered in the structural assembly simulations, the final model had a reasonable accuracy on both N- and C-terminal regions (**Fig 5F**), which resulted in a full-length model with a much higher TM-score (0.792) than that by either CEthreader/I-TASSER (0.578) or ResPRE/CNS pipelines (0.637). This example highlights again the advantage of contact-map and threading template combinations in structural assembly simulations, in particular for the cases when low-coverage templates are identified, where contact map predictions can be used to help model the threading-unaligned regions.

**Fig 6:**
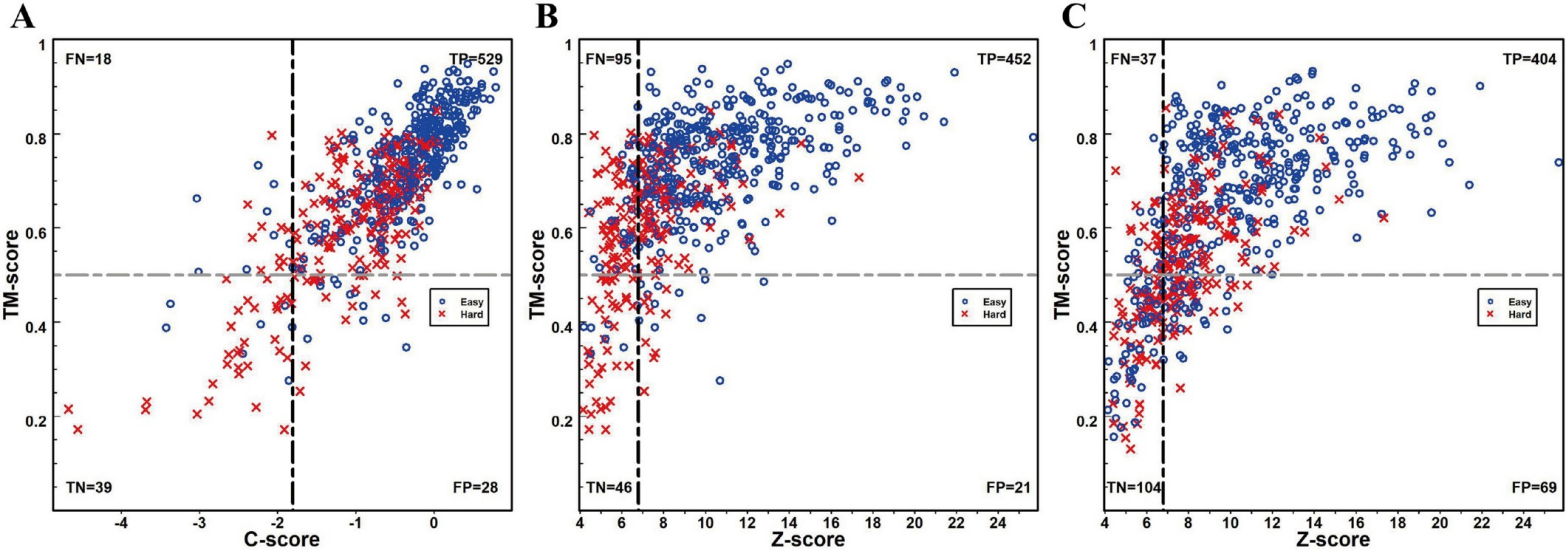
Accuracy estimation of predicted models using different score functions. (A) TM-score of the first C-I-TASSER model versus C-score defined by **Eq (8)**. (B) TM-score of the first C-I-TASSER model versus CEthreder threading Z-score defined by **Eq (7)**. (C) TM-score of the first threading template by CEthreader versus the Z-score. Blue circles and red crosses represent Easy and Hard targets respectively. The dashed vertical lines mark the score cutoffs that result in the maximum MCC value for distinguishing models with correct (TM-score >0.5) and incorrect (TM-score <0.5) folds, where values given in the sections indicate the number of points in each of the sections.

After predicting the final structure models of a target, it is important to estimate the accuracy of the models, e.g., determining whether a target was correctly folded (TM-score of the model ≥0.5) or not (TM-score <0.5). In **Fig 6A** and **6B**, we examined the correlations of the TM-score of the first C-I-TASSER model with two metrics of the C-I-TASSER C-score (defined in **Eq 8**in **METHODS**) and the threading Z-score (**Eq 7**). It was shown that the Pearson correlation coefficient (PCC) between TM-score and C-score (0.800) was much higher than that between TM-score and Z-score (0.579). If we selected a C-score cutoff of -0.18 to estimate the fold of final models, it would result in a maximum Matthews correlation coefficient (MCC) of 0.600, which was also much higher than the maximum MCC achieved by the threading Z-score (0.463). Here, it is noted that **Fig 6B** presented the TM-score of the C-I-TASSER models which might have only indirection relation with the threading Z-scores. To avoid the bias, we presented in **Fig 6C** the correlation data between the TM-score of the first threading template and the Z-score, where the PCC and the maximum MCC for the threading templates were 0.647 and 0.558, which were still lower than that from C-score/TM-score correlations (0.800 and 0.600). These results suggest that the C-score values by combining contact map, threading and MC simulation information should provide more accurate estimation on the final model quality compared to the widely threading Z-score.

## Discussion

In this study, we developed a new contact-guided threading approach, CEthreader, with the aim of significantly improving template-based structure modeling and fold-recognition for distant-homology proteins. The method first deduces residue-residue contact maps from multiple sequence alignments by integrating precision matrices with deep residual convolutional neural-network training. The predicted contact maps are then converted into single-body eigenvector products through eigen-decomposition, which enables the utilization of two-body contacts to guide the alignment construction and template selection using the Needleman-Wunch dynamic programming algorithm [46].

CEthreader was tested on two large-scale benchmark sets containing 614 non-redundant protein structures collected from the PDB and 539 domains selected from different families in the SCOPe database [36]. For the Hard proteins from Set-I that lacked homologous templates, CEthreader could detect the first-ranked templates with an average TM-score of 0.453, which was 9% or 45% higher than the best start-of-the-art threading approaches based exclusively on contact maps (map_align) or profiles (HHsearch), respectively, after excluding all templates with a sequence identity >30% to the query. Additionally, the number of correct templates with TM-scores >0.5 detected by CEthreader was 36% or 176% higher than the number identified by map_align or HHsearch. For Benchmark Set-II, CEthreader successfully recognized templates with the same SCOPe Fold as the query in 323 cases after excluding homologous templates in the same Superfamily, which was 20% or 114% higher than the number identified by EigenThreader or HHsearch, respectively. The detailed data analyses show that the major advantage of CEthreader lies in the utilization of contact map information in threading, which helps recognize the similarity between the global folds of the query and templates during the alignment procedure, even when the evolutionary relationship between them is weak; this is essential for modeling the structures of distant-homology proteins.

CEthreader was also compared to other profile- and contact-based threading methods for full-length 3D model constructions. Using the CEthreader alignments, MODELLER constructed full-length models with TM-scores >0.5 for 476 out of the 614 Benchmark Set-I proteins, which was 14% or 18% higher than the number of correct models built using either EigenThreader or HHsearch alignments, respectively. The performance of CEthreader-based template modeling can be further improved by coupling the template information with the predicted contact maps in the structural assembly simulations, for which the models constructed by CEthreader/C-I-TASSER demonstrated significantly higher TM-scores and folding success rates than those produced by CEthreader/I-TASSER or ResPRE/CNS. The data suggests that the interplay of contact-guided threading with predicted contact maps can further improve protein structure modeling and makes the CEthreader/C-I-TASSER approach go far beyond model reconstruction strategies built purely on contact map prediction or template recognition.

Despite the ability of CEthreader to recognize distant-homology templates, there is still room for further improvement. Currently, only positive eigenvalues have been considered in the eigen-decomposition process, which may result in a loss of information for some targets due to the omission of negative eigenvalues that are often important for precisely recovering the contact map matrix. Based on the statistics of the results of the Benchmark Set-I, for a typical protein, 47% of the eigenvalues are positive and 53% are negative (i.e., for a protein with 50 residues, there usually exist at least 23 positive eigenvalues) when performing eigen-decomposition utilizing native contact maps. Therefore, the inclusion of both positive and negative eigenvalues, which although may increase the implementation time of the program, should help further improve the contact-guided threading accuracy.

## Methods

CEthreader is a fold-recognition algorithm to identify similar-fold structures from the PDB under the guidance of predicted contact maps. The core part of the algorithm consists of contact map prediction, eigen-decomposition of the contact matrix, and contact-guided template search and selection, where the flowchart of the pipeline is depicted in **Fig 1**.

### Contact map prediction and the selection of residue-residue contacts

The contact map for a query sequence (with C_β_-C_β_ distances <8 Å) is predicted using the ResPRE method, which couples evolutionary precision matrices with deep residual neural networks [43]. Precision matrices are generated by ridge estimation of the inverse covariance matrix of the multiple sequence alignment, which is represented by an *L*×*L*×21×21 array of evolutionary couplings for a protein with *L* residues and *L*×*L* residue pairs. For each residue pair, the 21×21 coupling matrix is fed directly to the deep residual network (ResNet) [47], which is composed of 22 residual blocks where each block adds an identity map of the input to the output of the feedforward neural networks. ResPRE was trained using the Adam optimization algorithm [48] under the supervision of binary cross entropy loss and is implemented in PyTorch [49].

The ResPRE predicted contacts with sequence separation ≥5 are selected and used by CEthreader, where residue pairs located at the same helix with a separation of 4 are also considered in order to enhance helix alignment. The top *N*(= ∑_cr∈{*long*,*medium*,*short*}_*σ*_*cr*_*L*) predicted residue pairs, which are ranked by their confidence scores of prediction, are selected to form the final contact map, where *cr* refers to the *long*-, *medium*- and *short*-range contacts with sequence separation |*i*−*j*| ≥ 24, 23 ≥ |*i*−*j*| ≥ 12, and |*i*−*j*| ≤ 11, respectively. The parameters *σ*_*cr*_ were determined by maximizing the TM-score of CEthreader on a set of 905 training protein-pairs; these pairs were selected from the all-to-all alignments by TM-align [37] from a set of 335 non-redundant domains in SCOPe, where the TM-scores were roughly evenly-distributed in the range [0.5~0.95], i.e., with 241, 205, 190, 181 and 88 pairs with TM-scores in the range 0.5~0.6, 0.6~0.7, 0.7~0.8, 0.8~0.9 and 0.9~0.95, respectively. As shown in **S4A Fig**, the average TM-score of the CEthreader alignments is sensitive to *σ*_*cr*_, where the maximum value occurs near *σ*_*sum*_ = 2.51 (= ∑_*cr*_ *σ*_*cr*_ = 1.79+0.41+0.31), although the fraction of aligned contacts, *CMOq*, decreases monotonously with *σ*_*sum*_ (**S4B Fig**). In **S4C Fig**, we present the correlation data between the number of short, medium and long-range contacts and protein length calculated from 9,896 non-redundant SCOPe domains, where the linear regression with the fitted parameters coincides well with the experimental structure data.

In **S8 Table**, we list the performance of CEthreader on 905 training protein-pairs using contact maps from 18 different predictors. Although CEthreader was trained on the ResPRE contact maps, the data shows a strong correlation between the alignment performance and the accuracy of the contact map prediction from all different predictors. On average, ResPRE has the highest accuracy (84.6%) which also results in the highest TM-score (0.633) and *CMOq* (0.480) values used by CEthreader for the 905 training proteins.

### Eigen-decomposition of the contact maps

Contact maps can be represented by an *L*×*L* symmetric binary matrix, ***M***, in which residue pairs in contacts are designated as 1 and non-contacting pairs are set to 0. Based on the eigen-decomposition theory [50], we can infer that

$$M=\overset{L}{\underset{k=1}{\sum }}\lambda_{k}\vec{V_{k}}*{\vec{V_{k}}}^{T}$$ (1)

where *λ*_*k*_ represents the *k*-th eigenvalue of ***M***, and $\vec{V_{k}}={\left(v_{1,k},v_{2,k},\cdots ,v_{L,k}\right)}^{T}$ is the corresponding eigenvector. Since the contribution of each eigenvector depends on the absolute magnitude of the eigenvalue in **Eq (1)**, we reorder the eigenvalues in descending order ($\lambda_{1}\geq \lambda_{2}\geq \lambda_{3}\ldots \geq \lambda_{L}$), where the contact map can be approximated by considering only the largest *K* positive eigenvalues and associated eigenvectors:

$$M\approx \overset{K}{\underset{k=1}{\sum }}\lambda_{k}\vec{V_{k}}*{\vec{V_{k}}}^{T}$$ (2)

Here, we do not consider the negative eigenvalues because it introduces complex numbers into the following computation.

Based on **Eq (2)**, any pair of contacts between residues *i* and *j* can be written as

$$M_{i,j}\approx \left(\sqrt{\lambda_{1}}v_{i,1},\sqrt{\lambda_{2}}v_{i,2},\ldots ,\sqrt{\lambda_{K}}v_{i,K}\right)*{\left(\sqrt{\lambda_{1}}v_{j,1},\sqrt{\lambda_{2}}v_{j,2},\ldots ,\sqrt{\lambda_{K}}v_{j,K}\right)}^{T}$$ (3)

In this way, the contact profiles of the *i*- and *j*-th residues are described by the vectors of

$$\{\begin{array}{c} \vec{U_{i}}=\left(\sqrt{\lambda_{1}}v_{i,1},\sqrt{\lambda_{2}}v_{i,2},\ldots ,\sqrt{\lambda_{k}}v_{i,K}\right)\\ \vec{U_{j}}=\left(\sqrt{\lambda_{1}}v_{j,1},\sqrt{\lambda_{2}}v_{j,2},\ldots ,\sqrt{\lambda_{k}}v_{j,K}\right) \end{array}$$ (4)

This representation of contact maps using single-body profiles allows the integration of contact map information into a dynamic program alignment algorithm for fold-recognition (see **S2 Text** and **S5 Fig**in the SI for more details). In CEthreader, we set *K* = 7 as a trade-off between accuracy and speed for the alignment procedure (see **S3 Text** and **S6 Fig**).

### Contact-guided fold-recognition

The fold-recognition in CEthreader is performed by threading the query sequence through a non-redundant structure set taken from the PDB library. The contact-guided alignment score for aligning the *i-*th residue of the query with the *j-*th residue of the template protein is represented by

$$S_{cm+ss+prof}\left(i,j\right)=w_{1}*S_{cm}\left(i,j\right)+w_{2}*S_{prof}\left(i,j\right)+w_{3}*S_{ss}\left(i,j\right)+w_{4}$$ (5)

Here, the first term accounts for the contact map match between the query and template by (**S4 Text** and **S7 Fig** in SI):

$$S_{cm}\left(i,j\right)=\{\begin{array}{l} \frac{\vec{U_{i}}\cdot \vec{P_{j}}}{\text{max}{\left(\left|\vec{U_{i}}\left|,\right|\vec{P_{j}}\right|\right)}^{2}}\;\text{if}\;\left|\vec{U_{i}}\right|\neq \vec{0}\;and\;\left|\vec{P_{j}}\right|\neq \vec{0}\\ 0\;\left|\vec{U_{i}}\right|=\left|\vec{P_{j}}\right|=\vec{0} \end{array}$$ (6)

where $\vec{U_{i}}$ and $\vec{P_{j}}$ are the contact eigenvectors for the *i-*th residue of the query and the *j-*th residue of the template as defined by **Eq (4)**. This term helps to align the residues pairs from the query and template that have similar contact eigenvectors, and therefore enhances the match between the global contact maps of the two proteins (see **S8 Fig**). There are several previous studies trying to use a cosine function or a standard inner dot product function [28,29,31,33] as the scoring function when aligning the contact eigenvectors. However, cosine functions only consider the angle between two contact eigenvectors and ignore their lengths, which is suboptimal as both angle and length are important when deciding the similarity between two contact eigenvectors. Moreover, although the inner dot product function considers the lengths of the contact eigenvectors, it increases the contribution of the lengths of the contact eigenvectors when one of them is dramatically bigger than the other (see discussion in **S4 Text**). Indeed, based on our tests on the 905 query-template pairs (**S8 Table**), the scoring function defined by **Eq (6)** performs significantly better than the inner dot product-based scoring function in terms of query-template alignment TM-scores and CMOq values.

The second and third terms, *S*_*prof*_(*i*,*j*) and *S*_*ss*_(*i*,*j*), in **Eq (5)**, which are explained in **S5 Text,** take into account the sequence profile-to-profile and secondary structure alignment scores between the *i-*th residue of the query and the *j-*th residue of the template. The weighting parameters are *w*_1_ = 0.5, *w*_2_ = 0.4, *w*_3_ = 0.1, and *w*_4_ = 0.1, which were determined by maximizing the alignment TM-score for the 905 training protein-pairs described above.

The Needleman-Wunsch (NW) dynamic programming algorithm [46] is used to align two protein sequences, where an affine penalty scheme is utilized. For an *l* residue gap the gap penalty, *G* is determined by *G* = *g*_*o*_ + *g*_*e*_*l*, where *g*_o,e_ = *w*_1_*g*_*o*,*e*_(*cm*)+*w*_2_*g*_*o*,*e*_(*prof*)+*w*_3_*g*_*o*,*e*_(*ss*) with *g*_*o*_(*cm*) = *g*_*o*_(*prof*) = *g*_*o*_(*ss*) = −1.0, *g*_*e*_(*cm*) = *g*_*e*_(*prof*) = *g*_*e*_(*ss*) = −0.1 and *g*_*e*_(*ss*) = −0.077. These parameters were determined by optimizing the alignment performance on the 905 training protein pairs (see **S5 Text**). The gap penalty is used only for residues in the middle of the query and template sequences, and no penalty is applied to the terminal ends. Since changing the sign of ν_*i*,*k*_ and ν_*j*,*k*_ simultaneously gives the same *M*_*i*,*j*_ in **Eq (3)**, for a given eigen solution, there are 2^*K*^ different vectors all satisfying the eigen-decomposition equation. Thus, CEthreader needs to calculate $\sum_{k=1}^{K}2^{k}$ alignments for one query-template pair, where the alignment with the highest alignment score is selected.

In order to select the best alignment, *CMOq *is used to rank all the templates. Additionally, we can compute the Z-score based on *CMOq* to assess the quality of the template alignments with respect to the average, and further distinguish between good and bad templates. The Z-score is calculated by

$$\text{Z}-\text{score}\left(i\right)=\frac{CMOq\left(i\right)-\langle CMOq\rangle }{\sigma \left(CMOq\right)}$$ (7)

where *CMOq*(*i*) is the *CMOq* of the *i*-th template, and 〈*CMOq*〉 and *σ*(*CMOq*) are the average *CMOq* and standard deviation, respectively, across all templates for CEthreader.

### Structure assembly simulations based on an interplay of threading and contact map predictions

C-I-TASSER [44] (Contact-guided Iterative Threading ASSEmbly Refinement) is a fully-automated structure prediction pipeline that was used in 13^th^ community-wide Critical Assessment of Structure Prediction (CASP13) experiment. It is built on the I-TASSER [5] pipeline, but uses contact map information to enhance the accuracy of protein structure prediction. For a residue pair (*i* and *j*) that is predicted to be in contact, the restraint is added to the inherent I-TASSER potential by

$$E_{con}\left(d_{ij}\right)=\{\begin{array}{l} -U_{ij},\;\;\;\;\;\;\;\;\;\;\;\;\;\;\;\;\;\;\;\;\;\;\;\;\;\;\;\;\;\;\;\;\;\;\;\;\;\;\;\;\;\;\;\;\;\;\;\;\;\;d_{ij}<8Å\\ -\frac{1}{2}U_{ij}\left[1-sin\left(\frac{d_{ij}-\left(\frac{8+D}{2}\right)}{8}\pi \right)\right],\;\;\;8Å\leq d_{ij}<D\\ \frac{1}{2}U_{ij}\left[1+sin\left(\frac{d_{ij}-\left(\frac{D+80}{2}\right)}{\left(80-D\right)}\pi \right)\right],\;\;D\leq d_{ij}\leq 80Å\\ U_{ij},\;\;\;\;\;\;\;\;\;\;\;\;\;\;\;\;\;\;\;\;\;\;\;\;\;\;\;\;\;\;\;\;\;\;\;\;\;\;\;\;\;\;\;\;\;\;\;\;\;\;\;\;\;d_{ij}>80Å \end{array}$$ (8)

where *U*_*ij*_ is the contact probability between residue pair *i* and *j* predicted by ResPRE, *d*_*ij*_ is the C_β_-C_β_ distance between residue *i* and *j* in the simulation decoys, and *D* = 16 Å.

Following the simulations, the decoys from the simulation trajectories are clustered by SPICKER [51], where the conformations that are closest to cluster centroids are selected and refined by FG-MD [52] to generate the final top 5 structure models. The accuracies of the C-I-TASSER models are estimated through the confidence score (or C-score) which is calculated by

$$\text{C}-\text{score}=\text{ln}\left(\frac{M}{M_{tot}}\cdot \frac{1}{\langle RMSD\rangle }\cdot \left(\frac{\text{Z}-\text{score}\left(1\right)}{Z_{0}}+\frac{O\left(CM^{Q},CM^{M}\right)}{N\left(CM^{Q}\right)}\right)\right)$$ (9)

where *M* is the number of structure conformations in the SPICKER cluster; *M*_*tot*_ is the total number of C-I-TASSER structure decoys used for clustering; 〈*RMSD*〉 is the average RMSD of the decoys to the cluster centroid; Z – score(1) is the highest Z-score of the templates identified by CEthreader and *Z*_0_ = 6.8 is the corresponding Z-score cutoff for distinguishing between good and bad templates, which was determined based on the training dataset; *N*(*CM*^Q^) is the number of contacts used by CEthreader and *O*(*CM*^*Q*^, *CM*^*M*^) is the sum of probabilities provided by ResPRE for the overlapped contacts between the query and structure models.

## Supporting information

S1 Text The normalized number of effective sequences in an MSA. (PDF)

S2 Text Eigen-decomposition of a contact-map. (PDF)

S3 Text Optimization of the number of eigenvectors used by CEthreader. (PDF)

S4 Text Optimization of the scoring function used for aligning contact maps. (PDF)

S5 Text Optimization of the component scores used to match the secondary structure and sequence profiles of two proteins. (PDF)

S1 Table Summary of the threading alignments produced by CEthreader for the 614 test proteins in Benchmark Set-I. (PDF)

S2 Table Threading results by different methods for the 403 Easy targets from Benchmark Set-I. (PDF)

S3 Table Threading results for different methods using Benchmark Set-I separated based on sequence identity to the ResPRE training set. (PDF)

S4 Table Fold-recognition performance of CEthreader, HHsearch and MUSTER for 116 Fold families in Benchmark Set-II. (PDF)

S5 Table List of fold pairs in benchmark set-II with an average TM-score >0.4. (PDF)

S6 Table Summary of models built by MODELLER based on different threading methods for all 614 proteins in Benchmark Set-I. (PDF)

S7 Table Summary of models built by CEthreader/C-I-TASSER, CEthreader/I-TASSER and ResPRE/CNS for all 614 proteins in Benchmark Set-I. (PDF)

S8 Table CEthreader threading alignment results for the 905 query-template pairs using native contact maps or contact maps predicted using 18 different contact predictors. (PDF)

S1 Fig CEthreader template information for 211 Hard targets from Benchmark Set-I. (PDF)

S2 Fig CEthreader performance and time-cost using different numbers of eigenvectors in the greedy search step. (PDF)

S3 Fig Comparison of CEthreader’s performance to map_align and EigenThreader on Benchmark Set-I. (PDF)

S4 Fig Optimization of the number of predicted contacts to be used by CEthreader. (PDF)

S5 Fig The diagrammatic illustration of eigen-decomposition of a contact-map. (PDF)

S6 Fig Optimization of the number of eigenvectors used by CEthreader. (PDF)

S7 Fig Optimization of the contact map matching score *S*_*cm*_. (PDF)

S8 Fig Illustration that alignment of contact eigenvectors between query and template sequences results in the match of global contact maps for the two sequences. (PDF)
