# Supplementary material for: Detecting distant-homology protein structures by aligning deep neural-network based contact maps
Source: PLoS Comput Biol. 2019 Oct 17;15(10):e1007411. doi: 10.1371/journal.pcbi.1007411 (PMC6818797; doi:10.1371/journal.pcbi.1007411)
Supplement: S1 Text — (PDF) [file pcbi.1007411.s001.pdf]

Text S1. The normalized number of effective sequences (Neff) in an MSA

The depth of a multiple sequence alignment (MSA) can be measured by the normalized number of effective sequences (*Neff*):

$$Neff = \frac{1}{\sqrt{L}} \sum_{n=1}^{N} \frac{1}{1 + \sum_{m=1, m \neq n}^{N} I\left[S_{m,n} \geq 0.8\right]} \qquad (S1)$$

where L is the length of a query protein, N is the number of sequences in the MSA, and $S_{m,n}$ is the sequence identity between the m-th and n-th sequences. $I\left[S_{m,n} \geq 0.8\right]$ is equal to 1 if $S_{m,n} \geq 0.8$, or zero otherwise. Therefore, *Neff* is essentially equal to the number of non-redundant sequences (sequence identity<0.8) in the MSA normalized by the query length.
