# Supplementary material for: Detecting distant-homology protein structures by aligning deep neural-network based contact maps
Source: PLoS Comput Biol. 2019 Oct 17;15(10):e1007411. doi: 10.1371/journal.pcbi.1007411 (PMC6818797; doi:10.1371/journal.pcbi.1007411)
Supplement: S2 Text — (PDF) [file pcbi.1007411.s002.pdf]

Text S2. Eigen-decomposition of a contact-map

For a given protein P with length L, its contact map $\boldsymbol{M}$ can be represented by an $L \times L$ binary and symmetric matrix, where residues that are in contacts (C_β-C_β distance < 8 Å) are designated as 1 and non-contacting residues are set to 0. As the contact map $\boldsymbol{M}$ is a real-valued $L \times L$ symmetric matrix, it can be decomposed into L eigenvectors and their associated eigenvalues. Assuming that λ_i represents the i-th eigenvalue of $\boldsymbol{M}$ and $\vec{V_i} = (v_{1,i}, v_{2,i}, \ldots, v_{L,i})^T$ is the corresponding eigenvector, we have

$$\begin{cases} \boldsymbol{M}\vec{V_i} = \lambda_i \vec{V_i} \\ \boldsymbol{M} = V\Delta V^{-1} \end{cases} \qquad (S2)$$

where

$$\begin{cases} \mathrm{V} = \begin{bmatrix} v_{1,1} & \cdots & v_{1,L} \\ v_{2,1} & & v_{2,L} \\ \vdots & \ddots & \vdots \\ v_{L,1} & \cdots & v_{L,L} \end{bmatrix} = \left[\vec{V_1}, \vec{V_2}, \ \ldots\ldots, \vec{V_L}\right] \\ \Delta = \begin{bmatrix} \lambda_1 & \cdots & 0 \\ 0 & & 0 \\ \vdots & \ddots & \vdots \\ 0 & \cdots & \lambda_L \end{bmatrix} \end{cases} \qquad (S3)$$

Again, since $\boldsymbol{M}$ is a real-valued symmetric matrix, $V^{-1} = V^T$. Therefore, we can infer that

$$\begin{aligned} \boldsymbol{M} &= V\Delta V^{-1} = V\Delta V^T \\ &= \begin{bmatrix} v_{1,1} & \cdots & v_{1,L} \\ v_{2,1} & & v_{2,L} \\ \vdots & \ddots & \vdots \\ v_{L,1} & \cdots & v_{L,L} \end{bmatrix} \begin{bmatrix} \lambda_1 & \cdots & 0 \\ 0 & & 0 \\ \vdots & \ddots & \vdots \\ 0 & \cdots & \lambda_L \end{bmatrix} \begin{bmatrix} v_{1,1} & \cdots & v_{L,1} \\ v_{1,2} & & v_{L,2} \\ \vdots & \ddots & \vdots \\ v_{1,L} & \cdots & v_{L,L} \end{bmatrix} \\ &= \begin{bmatrix} v_{1,1} & \cdots & v_{1,L} \\ v_{2,1} & & v_{2,L} \\ \vdots & \ddots & \vdots \\ v_{L,1} & \cdots & v_{L,L} \end{bmatrix} \left(\sum_{i=1}^{L} \begin{bmatrix} 0 & \cdots & 0 \\ 0 & & 0 \\ \vdots & \lambda_i & \vdots \\ 0 & \cdots & 0 \end{bmatrix} \right) \begin{bmatrix} v_{1,1} & \cdots & v_{L,1} \\ v_{1,2} & & v_{L,2} \\ \vdots & \ddots & \vdots \\ v_{1,L} & \cdots & v_{L,L} \end{bmatrix} \\ &= \sum_{i=1}^{L} \lambda_i \vec{V_i} * \vec{V_i}^T \end{aligned} \qquad (S4)$$

Based on the above inference, the initial contact map $\boldsymbol{M}$ can be reconstructed by L eigenvalues and their associated eigenvectors, where eigenvalues are sorted in descending order. Typically, eigenvalues with greater absolute values are more important for reconstructing a contact map that is identical to the initial one. Thus, an approximate contact map can be constructed by considering only a few of its eigenvectors with large eigenvalues. Also, we do not consider the eigenvectors with negative eigenvalues because it requires introducing complex values into the following computation, which increases computational complexity significantly. In this way, the contact map $\boldsymbol{M}$ can be approximated by

$$
\begin{aligned}
\boldsymbol{M} &\approx \sum_{i=1}^{k} \lambda_i \vec{V_i} * \vec{V_i}^T \\
&= \begin{bmatrix} v_{1,1} & \cdots v_{1,k} & \cdots & 0 \\ v_{2,1} & v_{2,k} & \cdots & 0 \\ \vdots & \ddots & & \vdots \\ \vdots & \vdots & & \vdots \\ v_{L,1} & \cdots v_{L,k} & \cdots & 0 \end{bmatrix} \begin{bmatrix} \lambda_1 & \cdots & 0 \\ 0 & & 0 \\ \vdots & \lambda_k & \vdots \\ 0 & \cdots & 0 \end{bmatrix} \begin{bmatrix} v_{1,1} & \cdots & v_{1,L} \\ \vdots & & \vdots \\ v_{k,1} & \ddots & v_{k,L} \\ \vdots & \vdots & \vdots \\ 0 & \cdots & 0 \end{bmatrix} \\
&= \begin{bmatrix} v_{1,1} & \cdots v_{1,k} & \cdots & 0 \\ v_{2,1} & v_{2,k} & \cdots & 0 \\ \vdots & \ddots & & \vdots \\ \vdots & \vdots & & \vdots \\ v_{L,1} & \cdots v_{L,k} & \cdots & 0 \end{bmatrix} \begin{bmatrix} \sqrt{\lambda_1} & \cdots & 0 \\ 0 & & 0 \\ \vdots & \sqrt{\lambda_k} & \vdots \\ 0 & \cdots & 0 \end{bmatrix} \begin{bmatrix} \sqrt{\lambda_1} & \cdots & 0 \\ 0 & & 0 \\ \vdots & \sqrt{\lambda_k} & \vdots \\ 0 & \cdots & 0 \end{bmatrix} \begin{bmatrix} v_{1,1} & \cdots & v_{1,L} \\ \vdots & \vdots & \vdots \\ v_{k,1} & \ddots & v_{k,L} \\ \vdots & \vdots & \vdots \\ 0 & \cdots & 0 \end{bmatrix} \\
&= \begin{bmatrix} \sqrt{\lambda_1} v_{1,1} & \cdots \sqrt{\lambda_k} v_{1,k} & \cdots & 0 \\ \sqrt{\lambda_1} v_{2,1} & \sqrt{\lambda_k} v_{2,k} & \cdots & 0 \\ \vdots & \ddots & & \vdots \\ \vdots & \vdots & & \vdots \\ \sqrt{\lambda_1} v_{L,1} & \cdots \sqrt{\lambda_k} v_{L,k} & \cdots & 0 \end{bmatrix} \begin{bmatrix} \sqrt{\lambda_1} v_{1,1} & \cdots \sqrt{\lambda_k} v_{1,k} & \cdots & 0 \\ \sqrt{\lambda_1} v_{2,1} & \sqrt{\lambda_k} v_{2,k} & \cdots & 0 \\ \vdots & \ddots & & \vdots \\ \vdots & \vdots & & \vdots \\ \sqrt{\lambda_1} v_{L,1} & \cdots \sqrt{\lambda_k} v_{L,k} & \cdots & 0 \end{bmatrix}^T \\
&= \begin{bmatrix} \sqrt{\lambda_1} v_{1,1} & \cdots \sqrt{\lambda_k} v_{1,k} & \cdots & 0 \\ \sqrt{\lambda_1} v_{2,1} & \sqrt{\lambda_k} v_{2,k} & \cdots & 0 \\ \vdots & \ddots & & \vdots \\ \vdots & \vdots & & \vdots \\ \sqrt{\lambda_1} v_{L,1} & \cdots \sqrt{\lambda_k} v_{L,k} & \cdots & 0 \end{bmatrix} \begin{bmatrix} \sqrt{\lambda_1} v_{1,1} & \cdots \sqrt{\lambda_k} v_{1,k} & \cdots & 0 \\ \sqrt{\lambda_1} v_{2,1} & \sqrt{\lambda_k} v_{2,k} & \cdots & 0 \\ \vdots & \ddots & & \vdots \\ \vdots & \vdots & & \vdots \\ \sqrt{\lambda_1} v_{L,1} & \cdots \sqrt{\lambda_k} v_{L,k} & \cdots & 0 \end{bmatrix}^T
\end{aligned} \quad (S5)
$$

Assuming $C = \begin{bmatrix} \sqrt{\lambda_1} v_{1,1} & \cdots \sqrt{\lambda_k} v_{1,k} & \cdots & 0 \\ \sqrt{\lambda_1} v_{2,1} & \sqrt{\lambda_k} v_{2,k} & \cdots & 0 \\ \vdots & \ddots & & \vdots \\ \vdots & \vdots & & \vdots \\ \sqrt{\lambda_1} v_{L,1} & \cdots \sqrt{\lambda_k} v_{L,k} & \cdots & 0 \end{bmatrix}$, we can rewrite $\boldsymbol{M} \approx C * C^T$, and thus the initial contact between residue i and j can be approximated by

$$
M_{i,j} \approx \left(\sqrt{\lambda_1} v_{i,1}, \sqrt{\lambda_2} v_{i,2}, \ldots, \sqrt{\lambda_k} v_{i,k}, 0, \ldots, 0\right) * \left(\sqrt{\lambda_1} v_{j,1}, \sqrt{\lambda_2} v_{j,2}, \ldots, \sqrt{\lambda_k} v_{j,k}, 0, \ldots, 0\right)^T \quad (S6)
$$

Which means that we can describe the i-th and j-th residues of a protein by the contact eigenvectors $\vec{U_i} = \left(\sqrt{\lambda_1} v_{i,1}, \sqrt{\lambda_2} v_{i,2}, \ldots, \sqrt{\lambda_k} v_{i,k}, 0, \ldots, 0\right)$ and $\vec{U_j} = \left(\sqrt{\lambda_1} v_{j,1}, \sqrt{\lambda_2} v_{j,2}, \ldots, \sqrt{\lambda_k} v_{j,k}, 0, \ldots, 0\right)$, respectively. A protein can be represented by a k-dimensional contact eigenvector sequence.

The highest 7 eigenvalues and their corresponding eigenvectors can reconstruct a contact map that is comparable to the native contact map, as shown in **Fig. S5**. Additionally, the selection of 7 eigenvalues/eigenvectors is computationally efficient, as discussed later, and hence we consider the largest 7 eigenvalues and their corresponding eigenvectors to reconstruct the contact maps.

Two proteins with similar structures have similar contact maps, and thus have similar contact eigenvector sequences. Therefore, we perform the reverse process, where we first compare two contact eigenvector sequences in order to compare their corresponding structures. Using this technique, we decompose the native contact maps for the templates in our Benchmark Set-I and II, and obtain contact eigenvectors for each template. Since the goal of threading is to identify templates for query proteins that do not have experimentally solved structures, there is no native contact map information for the query proteins. Therefore, we use ResPRE [1] to predict each query protein's contact map starting from its sequence. The predicted contact maps are then decomposed to obtain the corresponding contact eigenvector sequences.

Using a semi-global dynamic programming algorithm, which does not penalize gaps in the terminal regions, we align the contact eigenvector sequences for the query and each template (**Fig. S5E**). It is noted that the sign of the eigenvectors has no influence on **Eq. (S4)**, i.e. $\vec{V_i} * \vec{V_i}^T = (-\vec{V_i}) * (-\vec{V_i}^T)$. Therefore, in order to consider all possible combinations, 2^K alignments are required, where K is the maximum number of eigenvectors used to reconstruct the contact maps. Moreover, when aligning K eigenvectors, we compute all alignments with 1 to K eigenvectors. As a result, the procedure must perform a total of $\sum_{k=1}^{K} 2^k = 2^{K+1} - 2$ alignments. For example, if we select K=7, we will evaluate $(2^1 + 2^2 + 2^3 + 2^4 + 2^5 + 2^6 + 2^7)$=254 alignments. In order to choose the best alignment, we utilize the *CMOq* index, which is defined as:

$$CMOq = \frac{O(CM^Q, CM^T)}{N(CM^Q)} \quad (S7)$$

where $N(CM^Q)$ is the number of contacts in the contact map for a query, and $O(CM^Q, CM^T)$ is the number of overlapped contacts between the aligned query and template. Note that the range of the *CMOq* is [0,1]. We observed a strong correlation (mean Pearson correlation coefficient = 0.945) between *CMOq* and TM-score, which is a widely used measurement to estimate the quality of the alignment between the query and template. Such a strong correlation indicates that the use of *CMOq* is a good index to choose the best alignment and template.

In order to evaluate the precision of predicted contact maps by different methods for all ranges, we calculate the *CMOacc* index using the following equation:

$$CMOacc = \frac{O(CM^{pred}, CM^{native})}{N(CM^{native})} \quad (S8)$$

where $N(CM^{native})$ is the number of native contacts for the query, and $O(CM^{pred}, CM^{native})$ is the number of overlapped contacts between the native contact map and predicted contact map. Here, we first sort predicted contacts in descending order of the confidence scores, and then select the top $N(CM^{native})$ predicted contacts. Note that the definition of *CMOacc* is similar to top L precision, except we replace L with the number of native contacts, and the value of *CMOacc* is close to 1 when the precision of the predicted contacts is 100%, while it is zero when the prediction precision is zero percent.

References

1. Li Y, Hu J, Zhang C, Yu D, Zhang Y. ResPRE: high-accuracy protein contact prediction by coupling precision matrix with deep residual neural networks. Bioinformatics. 2019.
