# Supplementary material for: Detecting distant-homology protein structures by aligning deep neural-network based contact maps
Source: PLoS Comput Biol. 2019 Oct 17;15(10):e1007411. doi: 10.1371/journal.pcbi.1007411 (PMC6818797; doi:10.1371/journal.pcbi.1007411)
Supplement: S3 Text — (PDF) [file pcbi.1007411.s003.pdf]

Text S3. Optimization of the number of eigenvectors used by CEthreader

Here, we justify the number of eigenvectors that we select in this study by analyzing the effect of the number of selected eigenvectors on the alignment accuracy and the time required for an alignment.

We tested two strategies: a fixed length model and a cumulative length model on the query-template pair dataset, where 335 query sequences were aligned to 905 templates, in order to determine the optimum number of eigenvectors. The fixed length model considered only *K* eigenvectors, while the cumulative length model considered 1 to *K* eigenvectors, where *K* ranged from 1 to 18. The TM-score based on the cumulative length model was always greater than that by the fixed length model, as shown in **Fig. S6A**. This is understandable because the search space for picking up the best alignment is relatively larger in the former model. Regardless of which model was being considered, the average TM-score increased as the number of selected eigenvectors increased. In particular, the average TM-score between the query and template increased rapidly from 0.533 to 0.633 when the selected number of eigenvectors increased from 1 to 7 when considering the cumulative length model. However, the improvement in the alignment quality was not very significant when more than 7 eigenvectors were used, since the average TM-score increased only an additional 2% when 8 to 18 eigenvectors were chosen. We note that, here, only contact information (S_{cm}) was used in the scoring function of the dynamic programming algorithm.

Although the alignment quality improved as the number of selected eigenvectors, *K*, was increased, the time complexity likewise increased logarithmically as the number of selected eigenvectors increased (**Fig. S6B**). For example, when we set *K*=7 in the cumulative model, the average time required to align a query sequence to the 905 templates was 1.55 hours ($e^{8.63}$ seconds). On the other hand, 6,710 hours (e^{17} seconds) were required when *K* was set to 18. While searching a query composed of 200 amino acids through the whole SCOPe database comprising 23,000 templates takes ~10 hours using *K*=7, it would require an enormous amount of time to perform threading with a higher value of *K*. Therefore, achieving the highest alignment quality level by increasing the number of eigenvectors is not feasible when only contact information is used in the scoring function.

When contact information was combined with the profile and secondary structure information (see **Text S5**), the average TM-score of the templates from a similar query-template pool was 0.66 based on *K*=7 using the cumulative model, as shown by the red circle in **Fig. S6A**. Additionally, the average TM-scores reached a plateau beyond 7 eigenvectors. Therefore, combining contact information with profile and secondary structure is more reasonable than increasing the number of eigenvectors. In order to further illustrate this point, we highlight an example from the alignment between the query Human ENPP4 with a Cleavable ATP-Analogue (SCOPe ID: d4le5a3) and the template S-adenosylmethionine synthetase (SCOPe ID: d1mxaa3) using different numbers of eigenvectors, where the results are presented in **Fig. S6C**. Here, the selection of 7 or 18 eigenvectors resulted in TM-scores of 0.81 or 0.90, respectively, when only contact information was used. On the other hand, with the combination of contacts, profile and secondary structure information, using *K*=7 eigenvectors resulted in a TM-score of 0.904. The inset in **Fig. S6C** shows that the overlap between the query and template, defined by $CMOq$ (**Eq. S7**), became flat when more than 7 eigenvectors were used, indicating optimum alignment between the query and template was achieved at *K*=7. Additionally, the eigenvalues did not decrease significantly when more than 7 eigenvectors were considered. Overall, the benchmark results indicate that the selection of 7 eigenvectors is sufficient and time efficient for reconstructing the query and

template contact maps and for generating a high-quality alignment between the two. Therefore, we select 7 eigenvectors to convert the two-body contact map information into a single-body potential, which is combined with the profile and secondary structure information for alignment and threading.
