# Supplementary material for: Detecting distant-homology protein structures by aligning deep neural-network based contact maps
Source: PLoS Comput Biol. 2019 Oct 17;15(10):e1007411. doi: 10.1371/journal.pcbi.1007411 (PMC6818797; doi:10.1371/journal.pcbi.1007411)
Supplement: S4 Text — (PDF) [file pcbi.1007411.s004.pdf]

Text S4. Optimization of the scoring function used for aligning contact maps

As mentioned before, we use a semi-global dynamic programming algorithm to align the contact eigenvectors for contact-map matching. The semi-global algorithm does not penalize gaps at the termini of the sequences, as gaps only incur penalties in the middle of the sequences. Here, we utilize an affine penalty schemes, $G_{cm} = g_o(cm) + g_e(cm) * l$, with gap opening penalty $g_o(cm) = -1.0$ and gap extension penalty $g_e(cm) = -0.1$, where l is the length of the gap in the contact-map (cm) alignment.

To align two contact-maps, Di Lena et al. [1] used the following dot product (dp) scheme:

$$S_{dp}(i,j) = \overrightarrow{U_i} \cdot \overrightarrow{P_j} = \sum_{l=1}^{k} \sqrt{\lambda_l} u_{il} \sqrt{\lambda_l} p_{jl} \qquad (S9)$$

where $\overrightarrow{U_i} = \left(\sqrt{\lambda_1} u_{i,1}, \sqrt{\lambda_2} u_{i,2}, \ldots, \sqrt{\lambda_k} u_{i,k}\right)$ is the contact eigenvector of the i-th residue of a query and $\overrightarrow{P_j} = \left(\sqrt{\lambda_1} p_{j,1}, \sqrt{\lambda_2} p_{j,2}, \ldots, \sqrt{\lambda_k} p_{j,k}\right)$ is the contact eigenvector of the j-th residue of the template.

In our study, we propose a revised version of the dot product scoring function, which shows an improved alignment performance, as discussed later. The modified dot product scoring function is written as:

$$S_{cm}(i,j) = \begin{cases} \dfrac{\overrightarrow{U_i} \cdot \overrightarrow{P_j}}{\max(|\overrightarrow{U_i}|, |\overrightarrow{P_j}|)^{\alpha}} & \text{if } \overrightarrow{U_i} \neq \vec{0} \; and \; \overrightarrow{P_j} \neq \vec{0} \\ 0 & \text{if } \overrightarrow{U_i} = \overrightarrow{P_j} = \vec{0} \end{cases} \qquad (S10)$$

Here, we use the max $(|\overrightarrow{U_i}|, |\overrightarrow{P_j}|)^{\alpha}$ to nomalize the dot product scoring function. In order to optimize the parameter α in the scoring function, we set it to a value ranging from [0.5, 4.0] at an interval of 0.1, and calculated the mean TM-score and mean *CMOq* from the alignment results based on different α values. As shown in **Fig. S7**, $\alpha \approx 2.0$ results in the largest *CMOq* and TM-score based on the gap penalty. Therefore, we selected α=2.0 in our final scoring function:

$$S_{cm}(i,j) = \begin{cases} \dfrac{\overrightarrow{U_i} \cdot \overrightarrow{P_j}}{\max(|\overrightarrow{U_i}|, |\overrightarrow{P_j}|)^{2}} & \text{if } \overrightarrow{U_i} \neq \vec{0} \; and \; \overrightarrow{P_j} \neq \vec{0} \\ 0 & \text{if } \overrightarrow{U_i} = \overrightarrow{P_j} = \vec{0} \end{cases} \qquad (S11)$$

The selection of α=2 has further mathematical significance, which is illustrated by the examples in **Fig. S7C**. As shown in the top left portion of **Fig. S7C**, two contact eigenvectors with lengths 2 and 10 are at an angle of 30^0. However, on the top right side of the figure, there are two contact eigenvectors with lengths 2 and 1.8, which are at a similar included angle. Based on the definition of the dot product $(\boldsymbol{S_{dp}})$ scoring function by Di Lena et al., the former group of vectors has a score of 17.3, while the latter group has a score of 3.06, indicating the contact eigenvector of length 2 tends to match with the contact eigenvector of length 10 during dynamic programming alignment. However, since the aim of our method is to align two similar contact eigenvectors, we should design a scoring function in a way that it gives a higher score to the second group of vectors than the first group in the example, since the second group of vectors are more similar. To this end, we designed an extended dot product $(\boldsymbol{S_{cm}})$ scoring function, which has the ability to scale different contact eigenvectors into a unit circle with a radius of 1 according to the larger contact eigenvector. Based on our

scoring function, the first and second groups have a score of 0.173 and 0.765, respectively, illustrating the scoring function has a better mathematical significance.

To further examine the performance of the normalized scoring function, we calculate the average $CMOq$ values and average TM-scores for 905 query-template pairs based on our scoring function and the one used by Di Lena et al. and present the findings in **Table S8**. Since the dataset is selected uniformly, we perform paired one-sided Wilcoxon single-ranked tests instead of Student t-tests to evaluate the significance of the difference between the dot product scoring function, S_{dp}, and our improved dot product scoring function, S_{cm}. Based on the same sets of contact map predictions, the average CMOq values (TM-scores) using S_{dp} and S_{cm} were 0.4165 (0.5853) and 0.4802 (0.6334), respectively, with a p-value of 1.54E-115 (2.48E-82). This indicates that the normalized dot product scoring function is more efficient than the simple dot product scoring function for detecting templates of similar folds as the query.

References

1. Di Lena P, Fariselli P, Margara L, Vassura M, Casadio R. Fast overlapping of protein contact maps by alignment of eigenvectors. Bioinformatics. 2010;26(18):2250-8. doi: 10.1093/bioinformatics/btq402.
