# Supplementary material for: Detecting distant-homology protein structures by aligning deep neural-network based contact maps
Source: PLoS Comput Biol. 2019 Oct 17;15(10):e1007411. doi: 10.1371/journal.pcbi.1007411 (PMC6818797; doi:10.1371/journal.pcbi.1007411)
Supplement: S5 Text — (PDF) [file pcbi.1007411.s005.pdf]

Text S5. Optimization of the component scores used to match the secondary structure and sequence profiles of two proteins

In addition to the contact-map alignment guided by **Eq. (S11)**, CEthreader includes two other energy terms that depend on sequence profile and secondary structure matching, which have been shown to be essential for improving alignment accuracy for both close- and distant-homology proteins [1]. Inspired by MUSTER, the normalized profile term is defined by [2]:

$$S_{prof}(i,j) = \sum_{k=1}^{20} P(i,k) * L(k,j)/max_{m,n}(|\sum_{k=1}^{20} P(m,k) * L(k,n)|), \qquad (S12)$$

where $P(i,k)$ is the frequency of the *k*-th amino acid at the *i*-th position in a multiple sequence alignment (MSA) obtained by PSI-BLAST search [3] of the query sequence through the NR database (ftp://ftp.ncbi.nlm.nih.gov/blast/db), where Henikoff [4] weighting is used to reduce the redundancy in the MSA. $L(k,j)$ is the log-odds profile (Position-Specific Substitution Matrix in PSI-BLAST with an *E*-value of 0.001) of the template sequence for the *k*-th amino acid at the *j*-th position. Here, we use a similar affine penalty scheme as used by the contact-based term, where the gap penalty $G_{prof} = g_o(prof) + g_e(prof) * l$, with gap opening penalty $g_o(prof) = -1.0$ and gap extension penalty $g_e(prof) = -0.1$, where *l* is the length of the gap in the profile (prof) guided alignments.

The secondary structure (SS) alignment term in CEthreader is defined by

$$S_{ss}(i,j) = \delta(s_i, s_j) \qquad (S13)$$

where s_i is the secondary structure of the *i*-th residue of the query as predicted by PSSpred [5] and s_j is the secondary structure of the *j*-th residue of the template as assigned by the DSSP program [6]. To align the query and template secondary structures, we follow a very similar protocol as MUSTER [2], where $S_{ss}(i,j)$ is equal to 0.093 if $s_i = s_j$, and -0.093 otherwise. Again we employ an affine penalty scheme, where the gap penalty $G_{ss} = g_o(ss) + g_e(ss) * l$, with gap opening penalty $g_o(ss) = -1.0$ and gap extension penalty $g_e(ss) = -0.077$, where l is the gap length. Moreover, no gaps are allowed inside the continuous secondary structure regions (helices and strands), i.e., with gap open and gap extension penalties of -100 in these regions.

In summary, the final multi-feature scoring function and gap penalties used in CEthreader can be written as:

$$\begin{cases} S_{cm+ss+prof}(i,j) = \omega_1 S_{cm}(i,j) + \omega_2 S_{prof}(i,j) + \omega_3 S_{ss}(i,j) + \omega_4 \\ g_o = \omega_1 g_o(cm) + \omega_2 g_o(prof) + \omega_3 g_o(ss) \\ g_e = \omega_1 g_e(cm) + \omega_2 g_e(prof) + \omega_3 g_e(ss) \\ \omega_1 + \omega_2 + \omega_3 = 1 \\ \omega_4 > 0 \end{cases} \qquad (S14)$$

Here, we utilize a weighting strategy for the three scoring function terms and gap penalties. Also, we set a bonus term for matching identical residues to each other. The optimized values of the four parameters are $\omega_1 = 0.5, \omega_2 = 0.4, \omega_3 = 0.1$ and $\omega_4 = 0.1$, based on the 905 query-template pairs dataset.

References

1. Zhang Y (2014) Interplay of I-TASSER and QUARK for template-based and ab initio protein structure prediction in CASP10. Proteins: Structure, Function, and Bioinformatics 82: 175-187.
2. Wu S, Zhang Y (2008) MUSTER: Improving protein sequence profile–profile alignments by using multiple sources of structure information. Proteins: Structure, Function, and Bioinformatics 72: 547-556.
3. S F Altschul, T L Madden, A A Schäffer, J Zhang, Z Zhang, et al. (1997) Gapped BLAST and PSI-BLAST: a new generation of protein database search programs. Nucleic Acids Res 25(17): 3389–3402.
4. Henikoff S, Henikoff JG (1994) Position-based sequence weights. Journal of Molecular Biology 243: 574-578.
5. Yan R, Xu D, Yang J, Walker S, Zhang Y (2013) A comparative assessment and analysis of 20 representative sequence alignment methods for protein structure prediction. 3: 2619.
6. Kabsch W, Sander C (1983) Dictionary of protein secondary structure: Pattern recognition of hydrogen-bonded and geometrical features. Biopolymers 22: 2577-2637.
