# Supplementary material for: Detecting distant-homology protein structures by aligning deep neural-network based contact maps
Source: PLoS Comput Biol. 2019 Oct 17;15(10):e1007411. doi: 10.1371/journal.pcbi.1007411 (PMC6818797; doi:10.1371/journal.pcbi.1007411)
Supplement: S1 Table — (PDF) [file pcbi.1007411.s006.pdf]

**Table S1.** Summary of the threading alignments produced by CEthreader for the 614 test proteins in Benchmark Set-I, where different component scores were used. $P$-values were calculated between $S_{cm+ss+prof}$ and the other score functions based on Wilcoxon signed-rank tests. $N_{st}$ is the number of cases with a TM-score >0.5 in each category.

| **Target type (# proteins)** | **Component scores** | **TM-score** | ***p*-value** | **RMSD** | **Coverage** | $N_{st}$ |
|---|---|---|---|---|---|---|
| Hard (211) | $S_{cm+ss+prof}$ | 0.453 | - | 9.531 | 0.875 | 80 |
| | $S_{cm}$ | 0.439 | 2.31E-03 | 9.815 | 0.873 | 71 |
| | $S_{ss+prof}$ | 0.284 | 7.75E-30 | 14.781 | 0.832 | 19 |
| Easy (403) | $S_{cm+ss+prof}$ | 0.687 | - | 4.795 | 0.909 | 365 |
| | $S_{cm}$ | 0.658 | 4.02E-22 | 5.270 | 0.907 | 339 |
| | $S_{ss+prof}$ | 0.656 | 1.14E-15 | 5.286 | 0.883 | 347 |
| All (614) | $S_{cm+ss+prof}$ | 0.607 | - | 6.423 | 0.898 | 445 |
| | $S_{cm}$ | 0.583 | 1.64E-19 | 6.832 | 0.895 | 410 |
| | $S_{ss+prof}$ | 0.528 | 2.14E-45 | 8.549 | 0.866 | 366 |
