# Supplementary material for: Detecting distant-homology protein structures by aligning deep neural-network based contact maps
Source: PLoS Comput Biol. 2019 Oct 17;15(10):e1007411. doi: 10.1371/journal.pcbi.1007411 (PMC6818797; doi:10.1371/journal.pcbi.1007411)
Supplement: S2 Table — (PDF) [file pcbi.1007411.s007.pdf]

Table S2. Threading results by different methods on the 403 Easy targets from Benchmark Set-I. P-values were calculated between the CEthreader alignment TM-scores and the other methods' TM-scores using pairwise one-sided Wilcoxon signed-rank tests; coverage is the number of aligned residues divided by length of the query; N_{st} denotes the number of targets whose templates were correctly identified with a TM-score >0.5.

**Methods**	**TM-score**	***p*-value**	**RMSD**	**Coverage**	N_{st}
**CEthreader**	0.687	-	4.795	0.909	365
**HHsearch**	0.682	2.24E-03	4.783	0.893	363
**MUSTER**	0.667	2.67E-11	5.171	0.895	348
**PPA**	0.656	1.14E-15	5.286	0.883	347
**map_align**	0.641	3.23E-23	5.724	0.902	328
**EigenThreader**	0.634	5.10E-39	5.670	0.904	324
**SAM-T99**	0.631	6.14E-29	5.020	0.839	330
**PROSPECT2**	0.618	1.46E-27	6.898	0.907	306
**FFAS03**	0.524	9.59E-49	7.350	0.810	258
