# Supplementary material for: Detecting distant-homology protein structures by aligning deep neural-network based contact maps
Source: PLoS Comput Biol. 2019 Oct 17;15(10):e1007411. doi: 10.1371/journal.pcbi.1007411 (PMC6818797; doi:10.1371/journal.pcbi.1007411)
Supplement: S3 Table — (PDF) [file pcbi.1007411.s008.pdf]

Table S3. Threading results for different methods using Benchmark Set-I separated based on sequence identity to the ResPRE training set. "<30%" corresponds to the subset of 149 Easy targets and 90 Hard targets which have sequence identities <30% to the ResPRE training set. "≥30%" represents the subset of 254 Easy targets and 121 Hard targets that have sequence identities ≥30% (and <40%) to the ResPRE training set. *P*-values were calculated between CEthreader alignment TM-scores and other methods' TM-scores using pairwise one-sided Wilcoxon signed-rank tests.

Target	Methods	*<30%*		*≥30%*	
		TM-score	*p*-value	TM-score	*p*-value
Easy targets	CEthreader	0.6729	-	0.6957	-
	HHsearch	0.6711	3.51E-02	0.6878	2.48E-03
	MUSTER	0.6548	7.49E-04	0.6738	2.59E-09
	PPA	0.6410	1.00E-05	0.6655	7.44E-12
	SAM-T99	0.6278	5.02E-10	0.6327	9.14E-21
	EigenThreader	0.6175	1.17E-15	0.6440	2.74E-25
	map_align	0.6073	8.43E-13	0.6610	1.93E-12
	PROSPECT2	0.6036	3.60E-12	0.6256	2.55E-17
	FFAS03	0.4838	3.75E-20	0.5478	2.04E-30
Hard targets	CEthreader	0.4372	-	0.4649	-
	EigenThreader	0.4031	1.86E-04	0.4208	1.00E-06
	map_align	0.4008	1.62E-03	0.4241	2.18E-04
	HHsearch	0.3115	1.70E-10	0.3137	7.98E-16
	MUSTER	0.2929	2.98E-12	0.3124	1.22E-17
	PPA	0.2618	2.53E-14	0.2999	1.59E-17
	PROSPECT2	0.2606	9.26E-16	0.2611	1.71E-21
	SAM-T99	0.2077	1.24E-15	0.2086	5.30E-21
	FFAS03	0.1833	3.83E-16	0.1936	1.99E-21
