# Supplementary material for: Detecting distant-homology protein structures by aligning deep neural-network based contact maps
Source: PLoS Comput Biol. 2019 Oct 17;15(10):e1007411. doi: 10.1371/journal.pcbi.1007411 (PMC6818797; doi:10.1371/journal.pcbi.1007411)
Supplement: S4 Table — (PDF) [file pcbi.1007411.s009.pdf]

Table S4. Fold-recognition performance of CEthreader, HHsearch and MUSTER on 116 Fold families in Benchmark Set-II. *Nq*: numbers of members in the SCOPe Fold families. *Nce, Nhh,* and *Nmu*: number of cases with a correct Fold template detected by CEthreader, HHsearch, and MUSTER, respectively, after filtering out all templates in the same Superfamily.

Fold	*Nq*	*Nce*	*Nhh*	*Nmu*	Fold	*Nq*	*Nce*	*Nhh*	*Nmu*
a.1	2	0	0	0	b.159	2	1	2	1
a.2	6	0	0	0	c.1	29	29	23	22
a.4	7	4	3	1	c.6	3	0	0	0
a.5	2	0	0	0	c.8	10	9	3	1
a.7	9	6	2	0	c.9	1	0	0	0
a.8	5	0	0	0	c.10	2	2	2	2
a.11	2	2	2	1	c.13	2	1	1	0
a.23	2	0	0	0	c.23	14	9	4	2
a.24	21	14	2	1	c.26	3	0	0	0
a.25	3	2	1	0	c.44	2	0	0	0
a.28	1	1	0	0	c.47	1	0	0	0
a.29	11	8	0	0	c.49	2	0	0	0
a.39	3	1	1	1	c.51	6	3	2	1
a.40	2	0	0	0	c.52	3	1	0	0
a.47	6	1	0	1	c.53	2	0	0	0
a.48	3	0	0	0	c.55	6	6	1	0
a.60	7	1	3	1	c.56	8	7	3	4
a.69	3	0	0	0	c.67	3	2	0	0
a.70	2	0	0	0	c.72	3	1	0	0
a.71	2	1	0	0	c.78	2	0	0	0
a.102	4	3	3	2	c.92	3	0	2	0
a.118	20	17	11	14	c.97	2	1	0	0
a.137	3	0	0	0	c.98	1	1	1	0
a.144	1	0	0	0	d.13	2	0	0	0
a.159	2	0	0	0	d.15	8	8	3	1
a.246	3	1	0	0	d.17	4	3	0	0
b.1	27	24	14	8	d.26	2	1	0	0
b.2	8	2	0	0	d.41	5	2	2	0
b.3	4	3	2	1	d.43	1	0	0	0
b.6	2	0	0	0	d.50	1	0	0	0
b.7	2	1	0	0	d.52	4	3	1	0
b.23	3	0	0	0	d.58	41	32	12	5
b.30	2	1	0	0	d.64	1	0	0	0
b.34	13	8	4	0	d.67	3	0	0	0
b.35	1	0	0	0	d.68	2	1	0	0
b.38	2	1	0	0	d.74	2	2	0	0
b.40	10	9	3	1	d.75	1	0	0	0
b.42	6	5	5	5	d.79	9	8	1	0
b.43	4	1	0	0	d.81	4	3	0	0
b.44	1	1	0	0	d.82	2	0	0	1
b.45	2	2	0	0	d.83	2	1	0	1
b.49	2	0	1	0	d.87	2	0	0	0
b.52	2	1	0	0	d.92	2	0	0	0
b.55	1	0	0	0	d.94	1	0	0	0
b.61	6	4	1	0	d.95	1	0	0	0
b.67	3	1	0	0	d.96	2	0	0	0
b.68	10	3	5	4	d.98	2	0	0	0

b.69	10	9	5	6	d.109	3	2	2	1
b.76	2	1	0	0	d.110	9	8	6	5
b.77	3	3	1	0	d.129	9	4	1	0
b.80	6	6	0	1	d.142	2	0	1	0
b.81	3	1	0	0	d.153	2	2	0	0
b.82	6	6	3	4	d.170	1	1	0	0
b.84	3	1	1	0	d.198	4	3	2	0
b.85	3	1	2	0	d.211	1	0	0	0
b.88	2	2	0	0	d.230	1	0	0	0
b.121	7	5	0	0	d.241	2	0	0	0
b.129	1	0	0	0	d.248	2	2	1	0
