# Supplementary material for: Detecting distant-homology protein structures by aligning deep neural-network based contact maps
Source: PLoS Comput Biol. 2019 Oct 17;15(10):e1007411. doi: 10.1371/journal.pcbi.1007411 (PMC6818797; doi:10.1371/journal.pcbi.1007411)
Supplement: S5 Table — (PDF) [file pcbi.1007411.s010.pdf]

Table S5. List of Fold pairs in Benchmark Set-II with an average TM-score >0.4.

Fold	Fold	TM-score	Fold	Fold	TM-score
a.2	a.7	0.578	a.7	a.24	0.474
a.2	a.8	0.506	a.7	a.29	0.481
a.2	a.24	0.535	a.7	a.47	0.583
a.2	a.29	0.550	a.7	a.102	0.456
a.2	a.47	0.597	a.7	a.118	0.423
a.2	a.102	0.485	a.8	a.24	0.492
a.2	a.118	0.449	a.8	a.29	0.512
a.2	c.1	0.408	a.8	a.47	0.455
a.2	c.56	0.411	a.8	a.102	0.400
a.2	d.129	0.405	a.24	a.29	0.452
a.7	a.8	0.459	a.24	a.47	0.446
a.29	a.47	0.443	c.1	c.8	0.401
a.29	a.60	0.405	c.1	c.23	0.427
a.102	a.118	0.427	c.1	d.79	0.402
b.1	b.2	0.518	c.23	c.51	0.421
b.1	b.3	0.464	c.23	c.56	0.444
b.1	b.121	0.428	c.51	c.56	0.425
b.2	b.3	0.419	c.56	d.198	0.405
b.2	b.121	0.412	d.41	d.58	0.439
b.61	d.17	0.419	d.41	d.129	0.416
b.61	d.129	0.404	d.52	d.58	0.417
b.68	b.69	0.600	d.58	d.129	0.418
