# Supplementary material for: Detecting distant-homology protein structures by aligning deep neural-network based contact maps
Source: PLoS Comput Biol. 2019 Oct 17;15(10):e1007411. doi: 10.1371/journal.pcbi.1007411 (PMC6818797; doi:10.1371/journal.pcbi.1007411)
Supplement: S6 Table — (PDF) [file pcbi.1007411.s011.pdf]

Table S6. Summary of models built by MODELLER based on different threading methods for all 614 proteins in Benchmark Set-I. P-values were calculated between the TM-scores of the models built using CEthreader alignments and other threading programs using pairwise one-sided Wilcoxon signed-rank tests. N_{st} represents the number of targets whose templates had a TM-score >0.5.

Target type (# proteins)	Methods	TM-score	p-value	RMSD (Å)	N_{st}
All (614)	CEthreader	0.628	-	4.999	476
	EigenThreader	0.585	3.05E-40	4.952	418
	map_align	0.584	7.26E-16	5.163	412
	HHsearch	0.571	1.77E-24	4.934	403
	MUSTER	0.564	1.08E-32	4.860	394
	PPA	0.548	2.43E-42	5.043	381
	PROSPECT	0.533	5.31E-49	5.018	352
	SAM-T99	0.510	1.64E-57	4.827	351
	FFAS03	0.425	1.25E-85	5.281	270
Easy (403)	CEthreader	0.708	-	4.749	372
	EigenThreader	0.656	1.62E-37	4.932	343
	map_align	0.663	2.03E-14	5.016	337
	HHsearch	0.698	4.42E-05	4.830	366
	MUSTER	0.694	6.61E-08	4.779	363
	PPA	0.677	2.58E-14	4.913	358
	PROSPECT	0.666	8.86E-15	5.024	339
	SAM-T99	0.658	7.63E-22	4.837	340
	FFAS03	0.541	7.39E-49	5.150	262
Hard (211)	CEthreader	0.476	-	5.475	104
	EigenThreader	0.449	4.21E-07	4.992	75
	map_align	0.432	6.04E-05	5.444	75
	HHsearch	0.330	3.72E-24	5.133	37
	MUSTER	0.316	4.68E-28	5.014	31
	PPA	0.301	2.01E-29	5.289	23
	PROSPECT	0.278	1.85E-34	5.006	13
	SAM-T99	0.228	3.32E-34	4.807	11
	FFAS03	0.205	9.68E-36	5.532	8
