# Supplementary material for: Detecting distant-homology protein structures by aligning deep neural-network based contact maps
Source: PLoS Comput Biol. 2019 Oct 17;15(10):e1007411. doi: 10.1371/journal.pcbi.1007411 (PMC6818797; doi:10.1371/journal.pcbi.1007411)
Supplement: S7 Table — (PDF) [file pcbi.1007411.s012.pdf]

Table S7. Summary of model quality for models built by CEthreader/C-I-TASSER, CEthreader/I-TASSER and ResPRE/CNS for all 614 proteins in Benchmark Set-I. P-values were calculated between the model TM-scores for CEthreader/C-I-TASSER and the other modeling approaches using pairwise one-sided Wilcoxon signed-rank tests. N_{st} represents the number of targets whose templates had a TM-score >0.5.

Target type (# proteins)	Methods	TM-score	p-value	RMSD (Å)	N_{st}
All (614)	CEthreader/C-I-TASSER	0.686	-	4.575	547
	CEthreader/I-TASSER	0.653	2.29E-21	4.769	502
	ResPRE/CNS	0.490	2.14E-91	4.707	312
Easy (403)	CEthreader/C-I-TASSER	0.734	-	4.319	384
	CEthreader/I-TASSER	0.721	6.36E-05	4.481	370
	ResPRE/CNS	0.518	1.19E-65	4.804	230
Hard (211)	CEthreader/C-I-TASSER	0.595	-	5.063	163
	CEthreader/I-TASSER	0.524	1.01E-21	5.319	132
	ResPRE/CNS	0.436	2.65E-26	4.523	82
