# Supplementary material for: Detecting distant-homology protein structures by aligning deep neural-network based contact maps
Source: PLoS Comput Biol. 2019 Oct 17;15(10):e1007411. doi: 10.1371/journal.pcbi.1007411 (PMC6818797; doi:10.1371/journal.pcbi.1007411)
Supplement: S8 Table — (PDF) [file pcbi.1007411.s013.pdf]

Table S8. CEthreader threading alignment results for the 905 query-template pairs using native contact maps or contact maps predicted using 18 different contact predictors [1-16]. Different contact predictors are sorted in descending order of contact accuracy, *CMOacc*. 'cm' and 'dp' correspond to alignments generated using the contact map score from **Eq. (6)** and the dot-product score from **Eq. (S9)** as proposed by Di Lena et al. [17], respectively.

Methods	CMOacc	TM-score		CMOq	
		cm	dp	cm	dp
**native**	1	0.6492	0.5975	0.5814	0.5021
**ResPRE**	0.8462	0.6334	0.5853	0.4802	0.4165
**DeepContact**	0.7634	0.6114	0.5724	0.4269	0.3775
**DeepCov**	0.6561	0.5830	0.5473	0.3726	0.3349
**NeBcon**	0.6244	0.5738	0.5412	0.3489	0.3140
**PconsC2**	0.5796	0.5632	0.5287	0.3276	0.2908
**MetaPSICOV**	0.5731	0.5700	0.5406	0.3350	0.3011
**GREMLIN**	0.3586	0.4931	0.4714	0.1956	0.1767
**CCMpred**	0.3579	0.4910	0.4676	0.1940	0.1752
**SVMcon**	0.3573	0.4684	0.4713	0.2242	0.2127
**BETAcon**	0.3319	0.4658	0.4712	0.2173	0.2074
**SVMSEQ**	0.3316	0.4606	0.4664	0.2234	0.2122
**plmDCA**	0.3160	0.4699	0.4567	0.1691	0.1550
**PSICOV**	0.3033	0.4750	0.4615	0.1738	0.1598
**NNcon**	0.2897	0.4342	0.4435	0.2002	0.1889
**PSpro.beta**	0.2633	0.4215	0.4374	0.1847	0.1754
**DNcon**	0.1986	0.4590	0.4599	0.2134	0.1987
**FreeContact**	0.1497	0.3127	0.3053	0.1287	0.1208
**PSpro**	0.1314	0.4277	0.4317	0.3266	0.3053

References

1. Li Y, Hu J, Zhang C, Yu D, Zhang Y (2019) ResPRE: high-accuracy protein contact prediction by coupling precision matrix with deep residual neural networks. Bioinformatics.
2. Liu Y, Palmedo P, Ye Q, Berger B, Peng J (2018) Enhancing Evolutionary Couplings with Deep Convolutional Neural Networks. Cell Systems 6: 65-74.e63.
3. Jones DT, Kandathil SM (2018) High precision in protein contact prediction using fully convolutional neural networks and minimal sequence features. Bioinformatics 34: 3308-3315.
4. Skwark MJ, Raimondi D, Michel M, Elofsson A (2014) Improved Contact Predictions Using the Recognition of Protein Like Contact Patterns. PLoS Comput Biol 10: e1003889.
5. Jones DT, Singh T, Kosciolek T, Tetchner S (2015) MetaPSICOV: Combining coevolution methods for accurate prediction of contacts and long range hydrogen bonding in proteins. Bioinformatics 31 (7): 999-1006.
6. Kamisetty H, Ovchinnikov S, Baker D (2013) Assessing the utility of coevolution-based residue-residue contact predictions in a sequence- and structure-rich era. Proc Natl Acad Sci U S A 110: 15674-15679.
7. Seemayer S, Gruber M, Söding J (2014) CCMpred—fast and precise prediction of protein residue–residue contacts from correlated mutations. Bioinformatics.
8. Cheng J, Baldi P (2007) Improved residue contact prediction using support vector machines and a large feature set. BMC Bioinformatics 8: 1-9.

9. Cheng J, Baldi P (2005) Three-stage prediction of protein β-sheets by neural networks, alignments and graph algorithms. Bioinformatics 21: i75-i84.
10. Wu S, Zhang Y (2008) A comprehensive assessment of sequence-based and template-based methods for protein contact prediction. Bioinformatics 24: 924-931.
11. Magnus E, Cecilia L, Yueheng L, Martin W, Erik A (2013) Improved contact prediction in proteins: using pseudolikelihoods to infer Potts models. Phys Rev E Stat Nonlin Soft Matter Phys 87.
12. Jones DT, Buchan DWA, Cozzetto D, Pontil M (2012) PSICOV: Precise structural contact prediction using sparse inverse covariance estimation on large multiple sequence alignments. Bioinformatics 28 (2): 184-190.
13. Tegge AN, Wang Z, Eickholt J, Cheng J (2009) NNcon: improved protein contact map prediction using 2D-recursive neural networks. Nucleic Acids Research 37: W515-W518.
14. Eickholt J, Cheng J (2013) A study and benchmark of DNcon: a method for protein residue-residue contact prediction using deep networks. BMC Bioinformatics 14: S12.
15. Kaján L, Hopf TA, Kalaš M, Marks DS, Rost B (2014) FreeContact: fast and free software for protein contact prediction from residue co-evolution. BMC Bioinformatics 15: 1-6.
16. He B, Mortuza SM, Wang Y, Shen HB, Zhang Y (2017) NeBcon: protein contact map prediction using neural network training coupled with naive Bayes classifiers. Bioinformatics 33: 2296-2306.
17. Di Lena P, Fariselli P, Margara L, Vassura M, Casadio R (2010) Fast overlapping of protein contact maps by alignment of eigenvectors. Bioinformatics 26: 2250-2258.
