# Supplementary material for: Detecting distant-homology protein structures by aligning deep neural-network based contact maps
Source: PLoS Comput Biol. 2019 Oct 17;15(10):e1007411. doi: 10.1371/journal.pcbi.1007411 (PMC6818797; doi:10.1371/journal.pcbi.1007411)
Supplement: S1 Fig — (PDF) [file pcbi.1007411.s014.pdf]

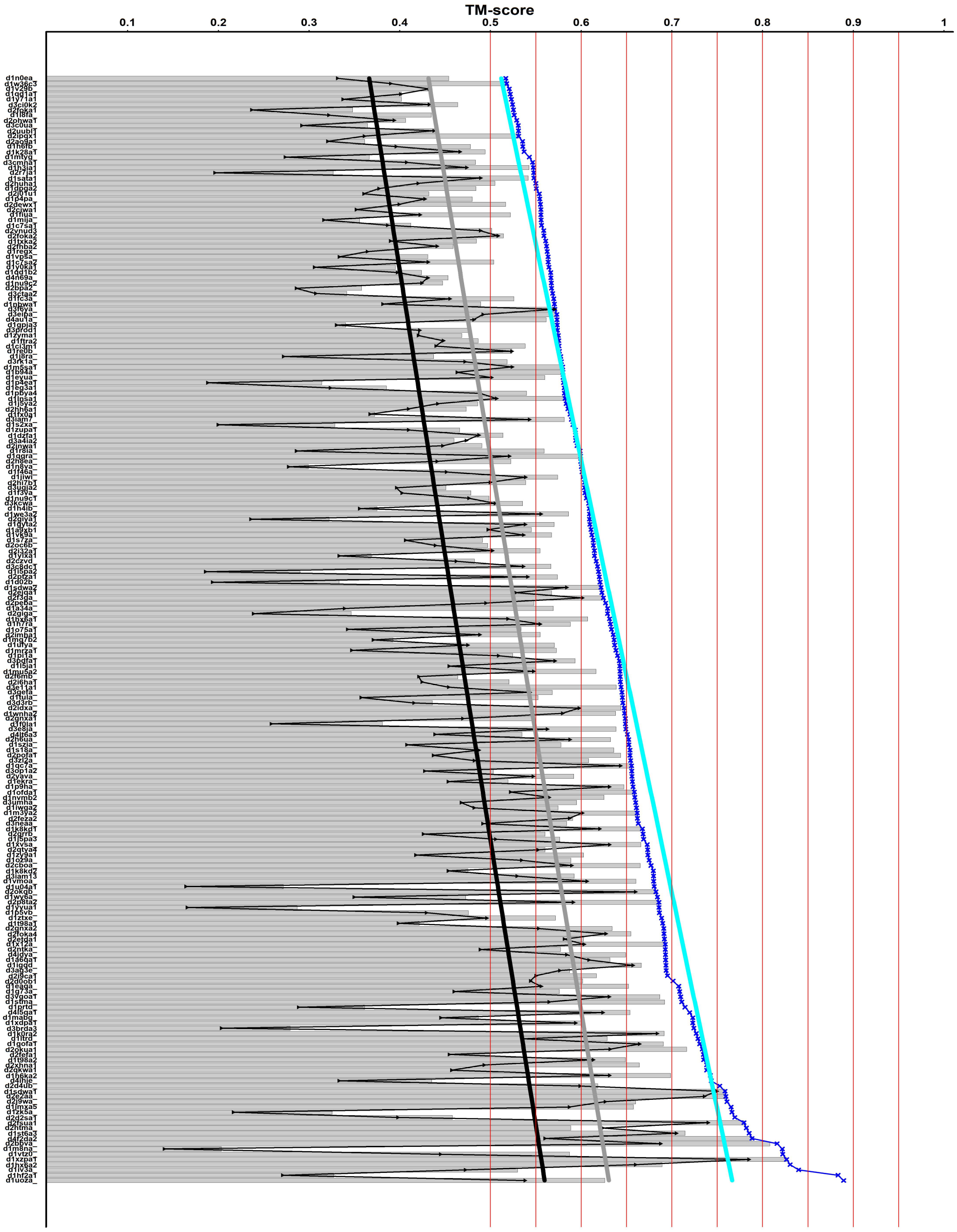

Figure S1. CEthreader template information for the 211 Hard targets from Benchmark Set-I. The blue cross points are the TM-scores of the best possible templates aligned to the corresponding target by the structure-based alignment method, TM-align; the gray bars mean the TM-scores of the first template detected by CEthreader but aligned by TM-align; the black triangles represents the TM-scores of the first templates detected and aligned by CEthreader. The linear regression is used to fit each corresponding set of TM-scores, where the fitted relationships for the cyan line, gray line and black line are y=0.501+ 0.001x, y=0.421+0.0008x, and y=0.356+0.0008x, respectively. Here we varied x from 1 to 211, which corresponds to the order of each target ranked by the TM-score of the best possible template.
