# Supplementary material for: Detecting distant-homology protein structures by aligning deep neural-network based contact maps
Source: PLoS Comput Biol. 2019 Oct 17;15(10):e1007411. doi: 10.1371/journal.pcbi.1007411 (PMC6818797; doi:10.1371/journal.pcbi.1007411)
Supplement: S2 Fig — (PDF) [file pcbi.1007411.s015.pdf]

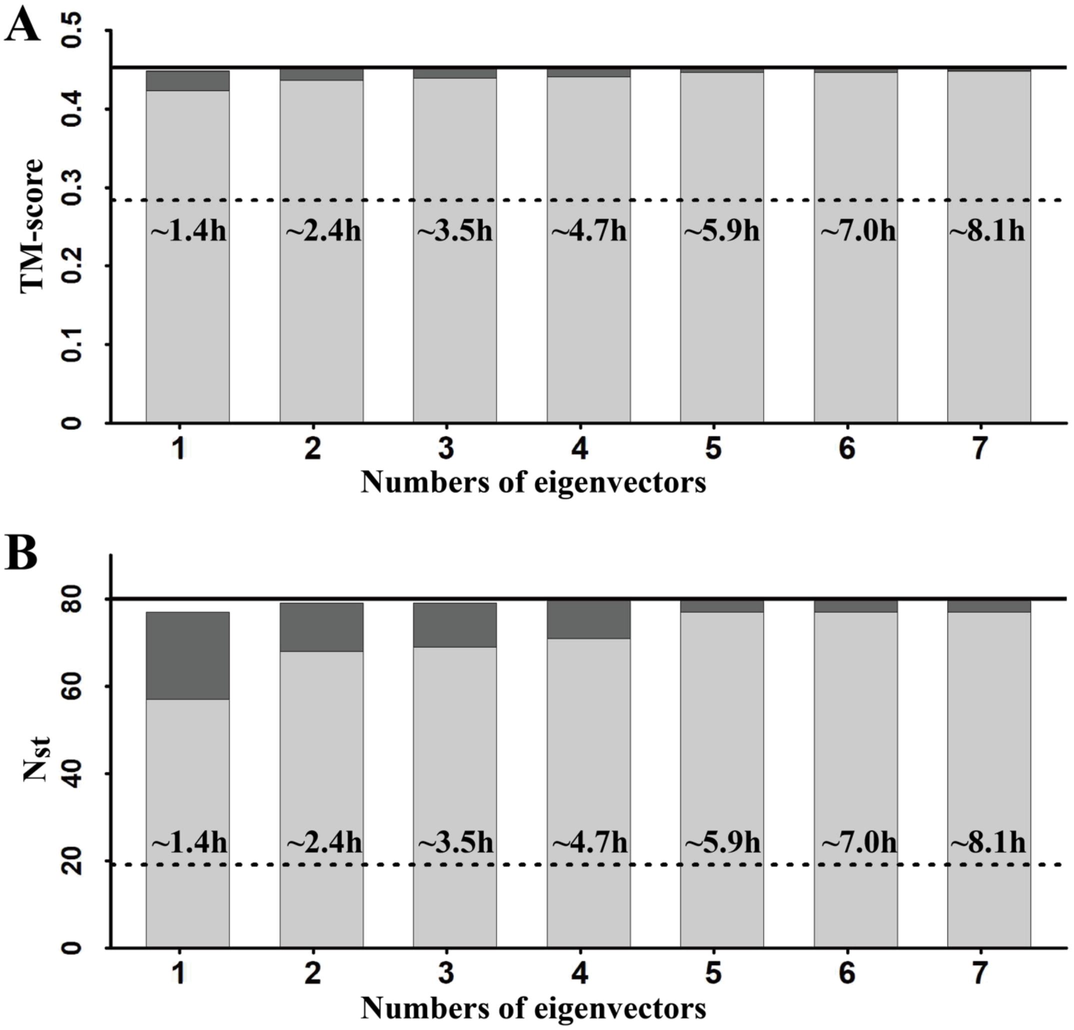

Figure S2. The performance and time-cost by CEthreader using different numbers of eigenvectors in the greedy search step. The height of the grey bar represents the average TM-score of the first template in (A) and the number of targets with identified templates that had a TM-score >0.5 in (B) based on the greedy searching strategy. The grey bar plus the black bar represents the results based on the hybrid searching strategy. The solid lines are the results based on enumerative searching with 7-dimensional eigenvectors using the scoring function $S_{cm+ss+prof}$, where the dotted line represents the results based only on profile and secondary structure information ($S_{ss+prof}$).
