# Supplementary material for: Detecting distant-homology protein structures by aligning deep neural-network based contact maps
Source: PLoS Comput Biol. 2019 Oct 17;15(10):e1007411. doi: 10.1371/journal.pcbi.1007411 (PMC6818797; doi:10.1371/journal.pcbi.1007411)
Supplement: S3 Fig — (PDF) [file pcbi.1007411.s016.pdf]

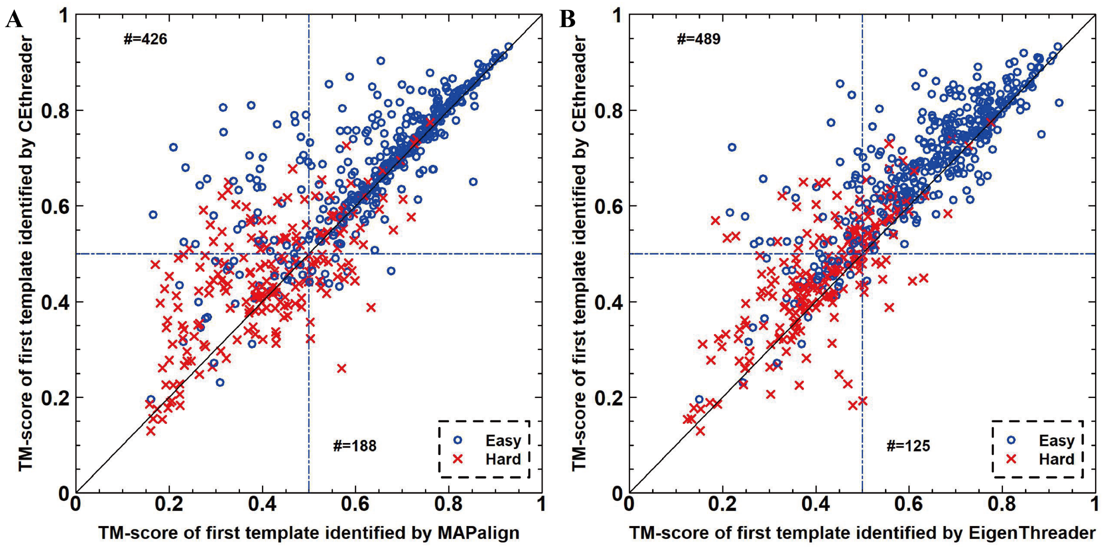

Figure S3. Comparison of CEthreader's performance to map_align and EigenThreader on Benchmark Set-I. (A) TM-score of the first template identified by CEthreader versus that by map_align. (B) TM-score of the first template identified by CEthreader versus that by EigenThreader.
