# Supplementary material for: Detecting distant-homology protein structures by aligning deep neural-network based contact maps
Source: PLoS Comput Biol. 2019 Oct 17;15(10):e1007411. doi: 10.1371/journal.pcbi.1007411 (PMC6818797; doi:10.1371/journal.pcbi.1007411)
Supplement: S4 Fig — (PDF) [file pcbi.1007411.s017.pdf]

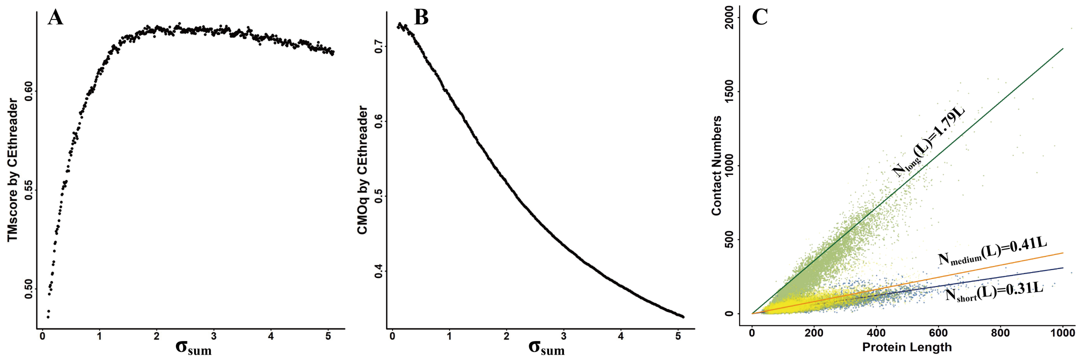

Figure S4. Optimization of the number of predicted contacts to be used by CEthreader. (A, B) TM-score and $CMOq$ as a function of σ_{sum} by CEthreader on 905 training protein pairs. (C) Correlation between the number of contacts and the sequence length for 9,896 non-redundant domains from the SCOPe database with a pair-wise sequence identity cutoff of <30%. The linear regression with fitted σ_{cr} parameters coincides well with the experimental structure data.
