# Supplementary material for: Detecting distant-homology protein structures by aligning deep neural-network based contact maps
Source: PLoS Comput Biol. 2019 Oct 17;15(10):e1007411. doi: 10.1371/journal.pcbi.1007411 (PMC6818797; doi:10.1371/journal.pcbi.1007411)
Supplement: S5 Fig — (PDF) [file pcbi.1007411.s018.pdf]

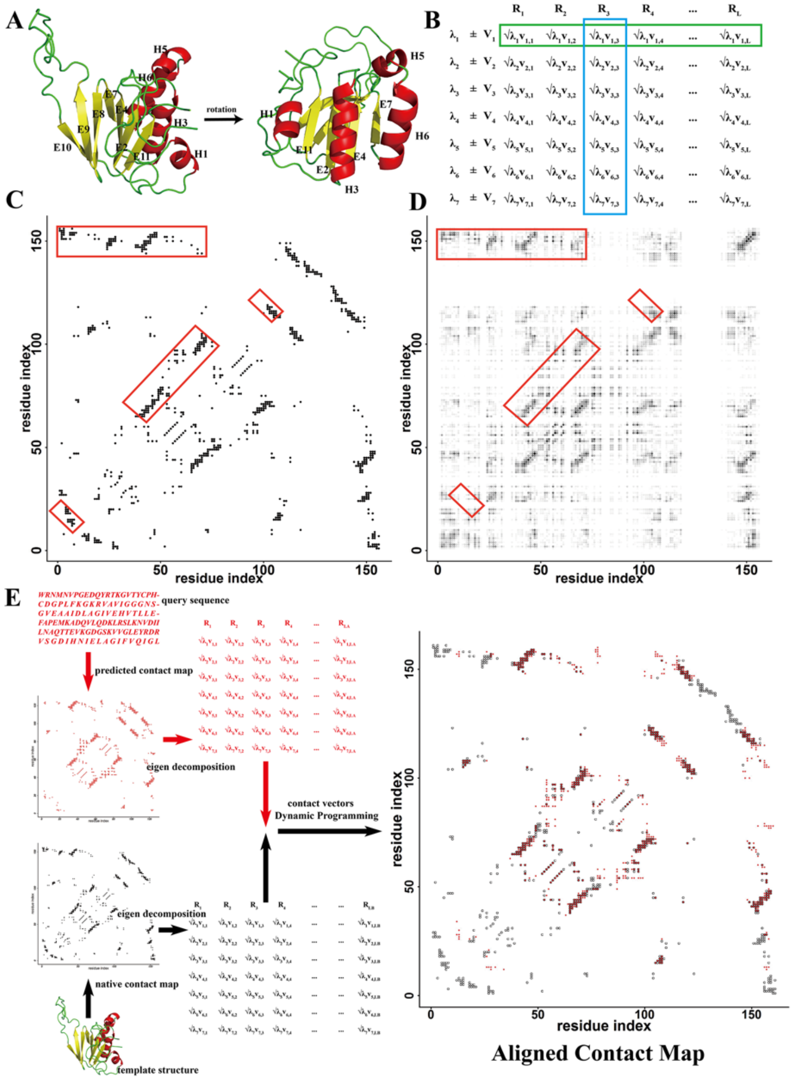

Figure S5. The diagrammatic illustration of eigen-decomposition of a contact-map. (A) 3D structure of the example from the trimethylamine dehydrogenase (SCOPe ID: d1o94a2). (B) the largest 7 eigenvalues and the corresponding eigenvectors decomposed from the native contact map; the green frame represents the *i*-th eigenvector, V_i, weighted by the square root of the corresponding eigenvalue, λ_i, and the blue frame describes the third contact eigenvector. (C) Native contact map for d1o94a2. (D) The contact map reconstructed using the largest 7 eigenvalues and their corresponding eigenvectors. The critical contacting residues in the red frames of (C) and (D) are very similar, indicating that the largest 7 eigenvalues and associated eigenvectors are sufficient for reconstructing the contact map. (E) Diagram of the query-template alignment. The upper left portion of the picture shows the predicted contact map for the query. The predicted contact map is then decomposed, resulting in the contact eigenvector sequence of the query. Similarly, we get the contact eigenvector sequence of the template from its native contact map, as shown in the lower left portion of the figure. Using a semi-global dynamic programming algorithm, we align the two contact eigenvector sequences that represent the query and template sequences. The picture on the right shows the overlapped contact maps for the query (red) and template (black).
