# Supplementary material for: Detecting distant-homology protein structures by aligning deep neural-network based contact maps
Source: PLoS Comput Biol. 2019 Oct 17;15(10):e1007411. doi: 10.1371/journal.pcbi.1007411 (PMC6818797; doi:10.1371/journal.pcbi.1007411)
Supplement: S6 Fig — (PDF) [file pcbi.1007411.s019.pdf]

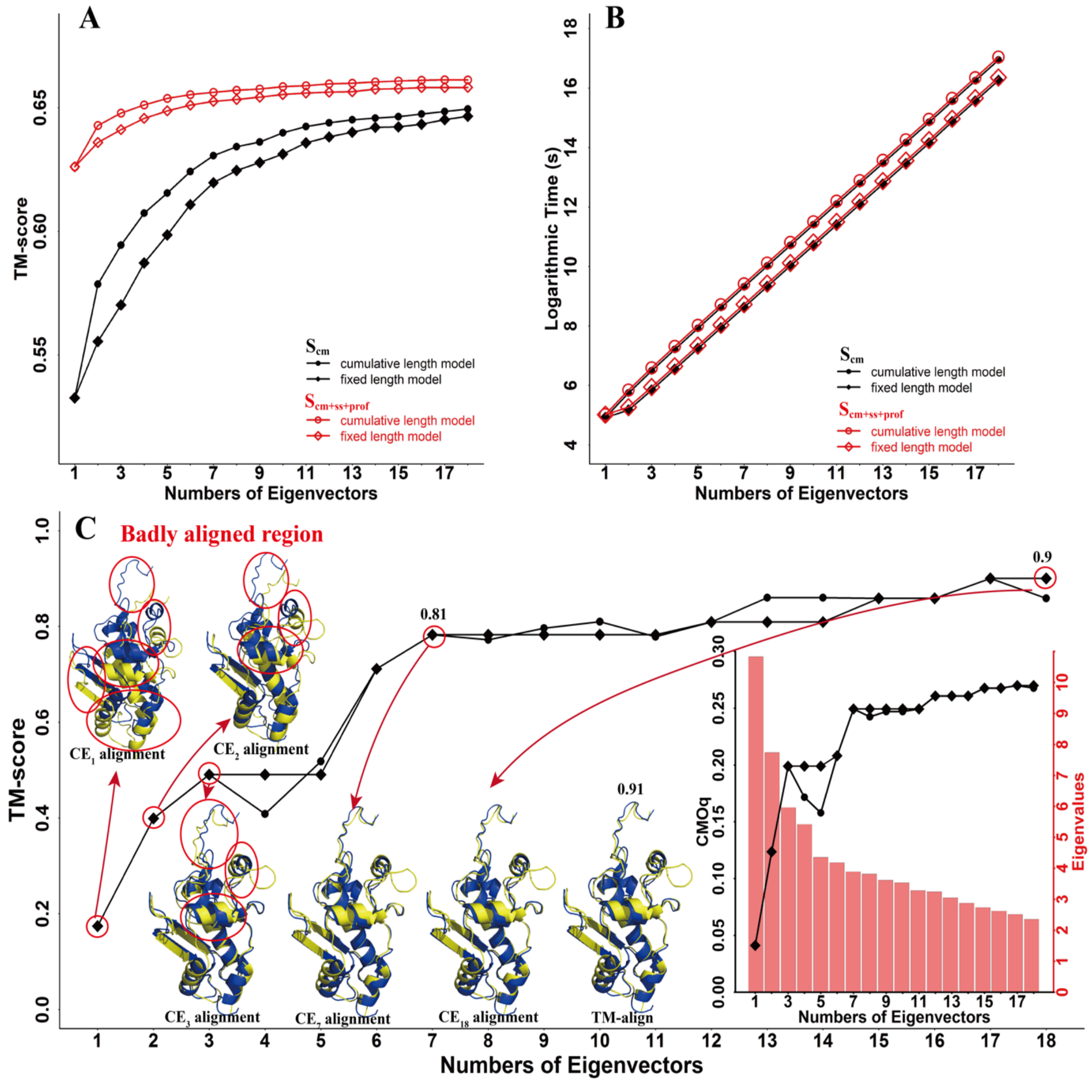

Figure S6. Optimization of the number of eigenvectors used by CEthreader. (A) The average alignment TM-score versus the number of eigenvectors used by CEthreader. Results based on contact information (S_{cm}) are shown using black circles (cumulative length model) and squares (fixed length model), while those based on the combination of contact, secondary structure, and profile information ($S_{cm+ss+prof}$) are highlighted using red circles (cumulative length model) and squares (fixed length model). (B) The logarithmic time required by CEthreader as a function of the number of eigenvectors. (C) TM-score as a function of the number of eigenvectors for the example protein pairs. The Inset shows *CMOq* (black points) and the eigenvalues (red bars) using various numbers of eigenvectors for the example. The superimposed structures of the template from S-adenosylmethionine synthetase (SCOPe ID: d1mxaa3) obtained using different numbers of eigenvectors onto the query Human ENPP4 with a Cleavable ATP-Analogue (SCOPe ID: d4lr5a3) are shown, where badly aligned regions are highlighted with red circles.
