# Supplementary material for: Detecting distant-homology protein structures by aligning deep neural-network based contact maps
Source: PLoS Comput Biol. 2019 Oct 17;15(10):e1007411. doi: 10.1371/journal.pcbi.1007411 (PMC6818797; doi:10.1371/journal.pcbi.1007411)
Supplement: S7 Fig — (PDF) [file pcbi.1007411.s020.pdf]

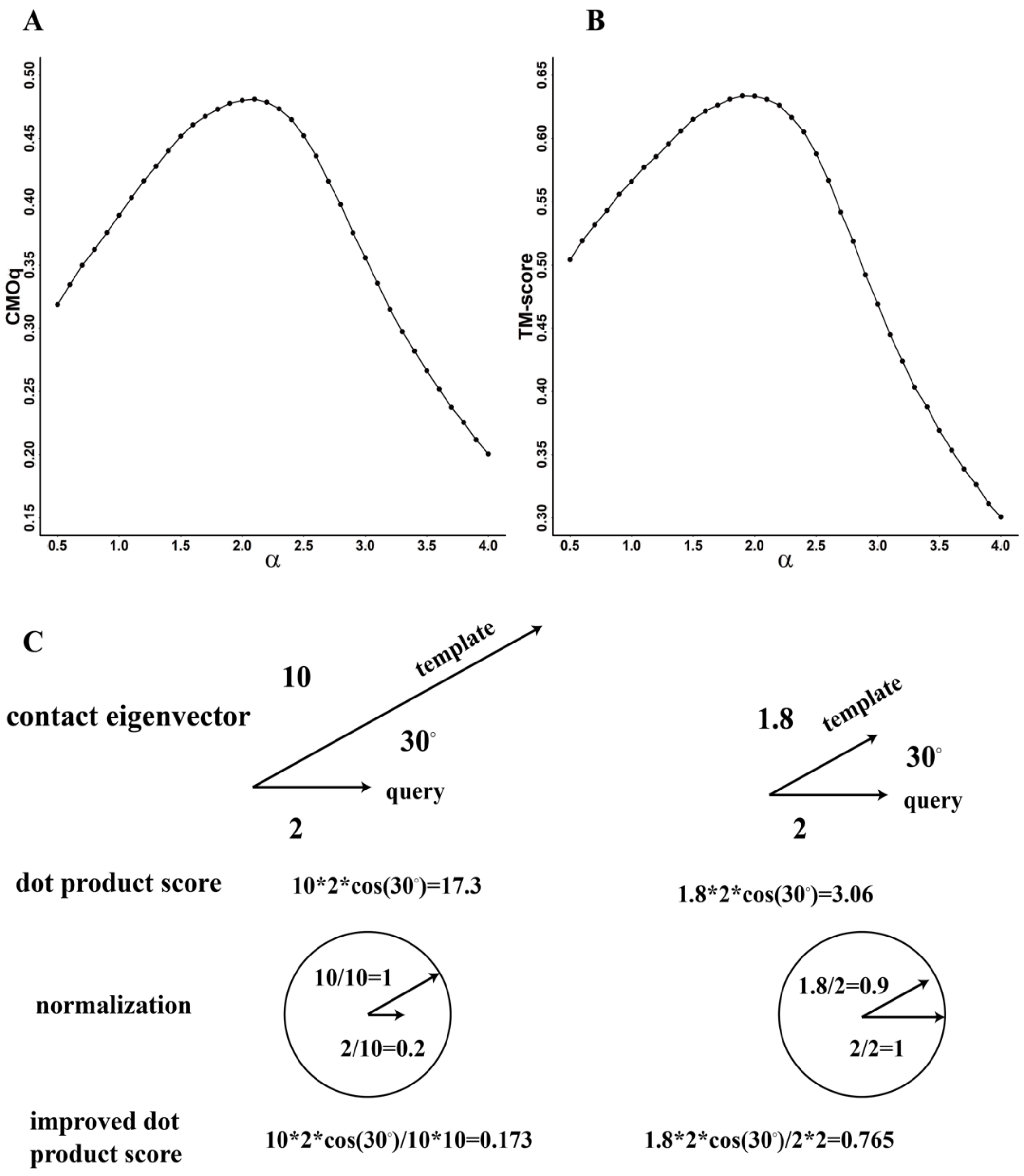

Figure S7. Optimization of the contact map matching score S_{cm}. Based on 905 training query-template pairs, we calculated the average TM-score and average $CMOq$ from the alignment results using different α values in **Eq. (S10)**. (A, B) Average $CMOq$ and TM-score as a function of α. (C) The comparison of dot product scoring function S_{dp} (top panel) from **Eq. (S9)** and the improved dot product scoring function S_{cm} (bottom panel) from **Eq. (S10)**.
