# Supplementary material for: Detecting distant-homology protein structures by aligning deep neural-network based contact maps
Source: PLoS Comput Biol. 2019 Oct 17;15(10):e1007411. doi: 10.1371/journal.pcbi.1007411 (PMC6818797; doi:10.1371/journal.pcbi.1007411)
Supplement: S8 Fig — (PDF) [file pcbi.1007411.s021.pdf]

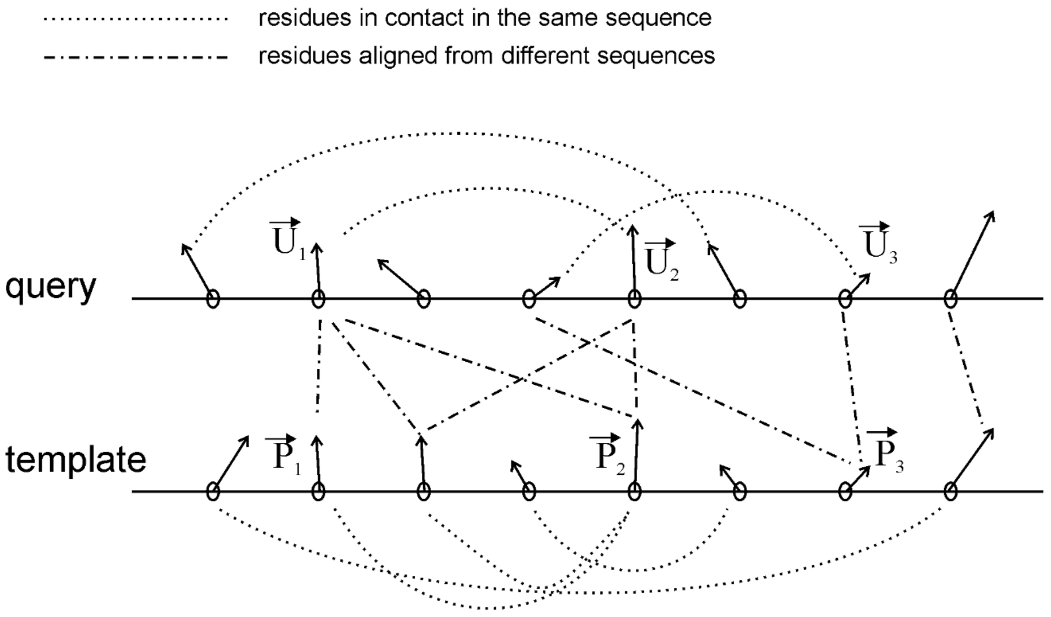

Figure S8. Illustration that alignment of contact eigenvectors between query and template sequences results in the match of global contact maps for the two sequences. Contact eigenvectors of individual residues are represented by the vectors along the sequences. Dotted lines connect the residues that are in contact in the same sequence, while dotted-dashed lines connect those residue pairs across the query and template chains that have high $S_{cm}(i,j)$ scores calculated using **Eq. (6)**. Although the contact vectors are defined by the contact matrix for the same sequence by **Eqs. (3)** and **(4)**, the picture shows that the alignment of $(\overrightarrow{U_i}, \overrightarrow{P_j})$ across query and template sequences can enhance the match of the global contact maps between them. In the illustrative example, when the three residue pairs between the query and template are well-aligned, i.e., $\overrightarrow{U_1} \cdot \overrightarrow{P_1} \sim \overrightarrow{U_2} \cdot \overrightarrow{P_2} \sim \overrightarrow{U_3} \cdot \overrightarrow{P_3} \sim 1$, the relationship between $\overrightarrow{U_1}$, $\overrightarrow{U_2}$ and $\overrightarrow{U_3}$, no matter if they are in contact ($\overrightarrow{U_1} \cdot \overrightarrow{U_2} = 1$) or not ($\overrightarrow{U_1} \cdot \overrightarrow{U_3} \ll 1$), will be similar to the relationship between $\overrightarrow{P_1}$, $\overrightarrow{P_2}$ and $\overrightarrow{P_3}$, i.e., the contact maps of the query and template will be similar.
